# Passive Membrane Transport Analysis of Drug Mixtures

**DOI:** 10.1021/acs.analchem.5c02264

**Published:** 2025-08-05

**Authors:** Robert Strutt, Simon F. Berlanda, Petra S. Dittrich

**Affiliations:** † Department of Biosystems Science and Engineering, 27219 ETH Zürich, Schanzenstrasse 44, 4056 Basel, Switzerland

## Abstract

Membrane transport is fundamental to biological cells and is a major hurdle in the rational design of pharmaceuticals. To measure membrane transport in vitro, most methods focus on simple diffusion of a single analyte across nonbiomimetic interfaces. Membrane engineering has facilitated novel strategies for the reconstitution, characterization, and application of biomimetic membranes. Herein, we define drug mixture analysis, an in vitro, label-free HPLC-MS, droplet interface bilayer (DIB) method, to assess membrane transport of drug mixtures and delineate simultaneous transport mechanisms. We use our method to classify the permeability of drugs from a structurally diverse FDA-approved library. This deep analysis uncovered correlation between determined permeability classifiers and drug properties such as the hydrophobic retention time, hydrogen bond donor count, lipophilicity, and predicted gut absorption. Across higher mimetic membranes, passive transport was quantified under physiologically relevant variables such as pH, temperature, lipid composition, and, in the presence of proteins, the coexistence of facilitated diffusion and simple diffusion. Our results show that DIBs are physiologically relevant interfaces for investigating membrane transport mechanisms relevant to artificial cell systems and drug screening.

## Graphical abstract

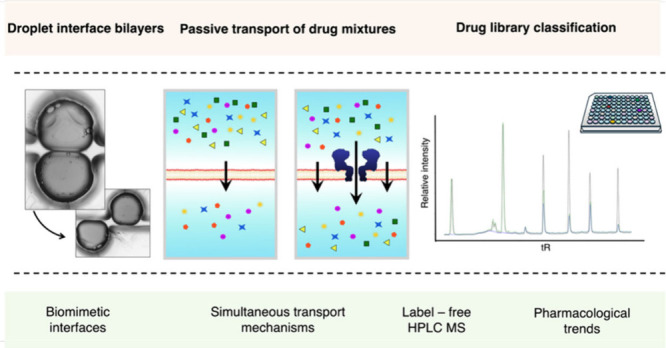

## Introduction

Most small molecule drugs must cross biological membranes since many drug targets are intracellular.^1–3^ A defining feature of membranes is their simultaneous facilitation of multiple transport mechanisms. As an example, consider a drug that must cross intestinal enterocyte cells and then accumulate intracellularly in a target cell. The drug may overcome the plasma membrane via passive transport (simple diffusion and facilitated diffusion), via active transport, via endocytosis, or via other mechanisms.^4,5^ Net membrane transport emerges through a balance of the influx and efflux rates contributed via these mechanisms. Due to local chemical and genetic heterogeneity, transport mechanism and the net transport rate vary across biological membranes.^6–9^ This coexistence of transport mechanisms combined with the massive scope of druggable chemical space has made the development of generalizable, predictive models highly challenging.^10,11^ The contentiousness of this research area has been evidenced recently through critique of the physiological relevance of simple diffusion in net drug transport.^12,13^ While there is a subset of mechanisms and processes, simple diffusion predominantly occurs from solvation into and desolvation from the lipids of biological membranes. Rates of simple diffusion have widely been measured in vitro revealing interesting trends,^14–21^ however, the relevance of these measurements is proportional to how physiologically relevant the in vitro interface is.^17,22–25^ New technologies are thereby required to reliably assess distinct drug transport mechanisms.

Droplet interface bilayers (DIBs) form spontaneously through the contact of water in oil droplets stabilized by lipid monolayers (Figure 1a). DIBs are commonly used as a chassis for artificial cell engineering and have formed the basis for diverse biomimetic structures and analytical techniques.^26–28^ They are especially well-suited to form geometrically defined compartments where membrane transport can be controlled and biological reactions within and across compartments may be well-simulated and studied.^29–32^ For example, we recently engineered a biohybrid system where encapsulation of living bacteria in a droplet network simulated treatment of an intracellular infection.^28^ A bilayer is fundamentally more similar to a biological membrane than the water–oil–water emulsion of a parallel artificial membrane permeation assay (PAMPA), which can exclusively measure simple diffusion.^33,34^ Several groups have reported label-free measurement of drug simple diffusion across DIBs or bilayer interfaces.^17,28,30,34,35^ The majority of these in situ analytical methodologies are constrained to assay highly concentrated, singular drug targets and are therefore limited in assessing biologically representative solutions containing multiple solutes or differentiating transport mechanisms. While previous results have indicated that DIBs are valuable models, additional benchmarking against pharmaceutical understanding of membrane transport and drug uptake is required.

**Figure 1:**
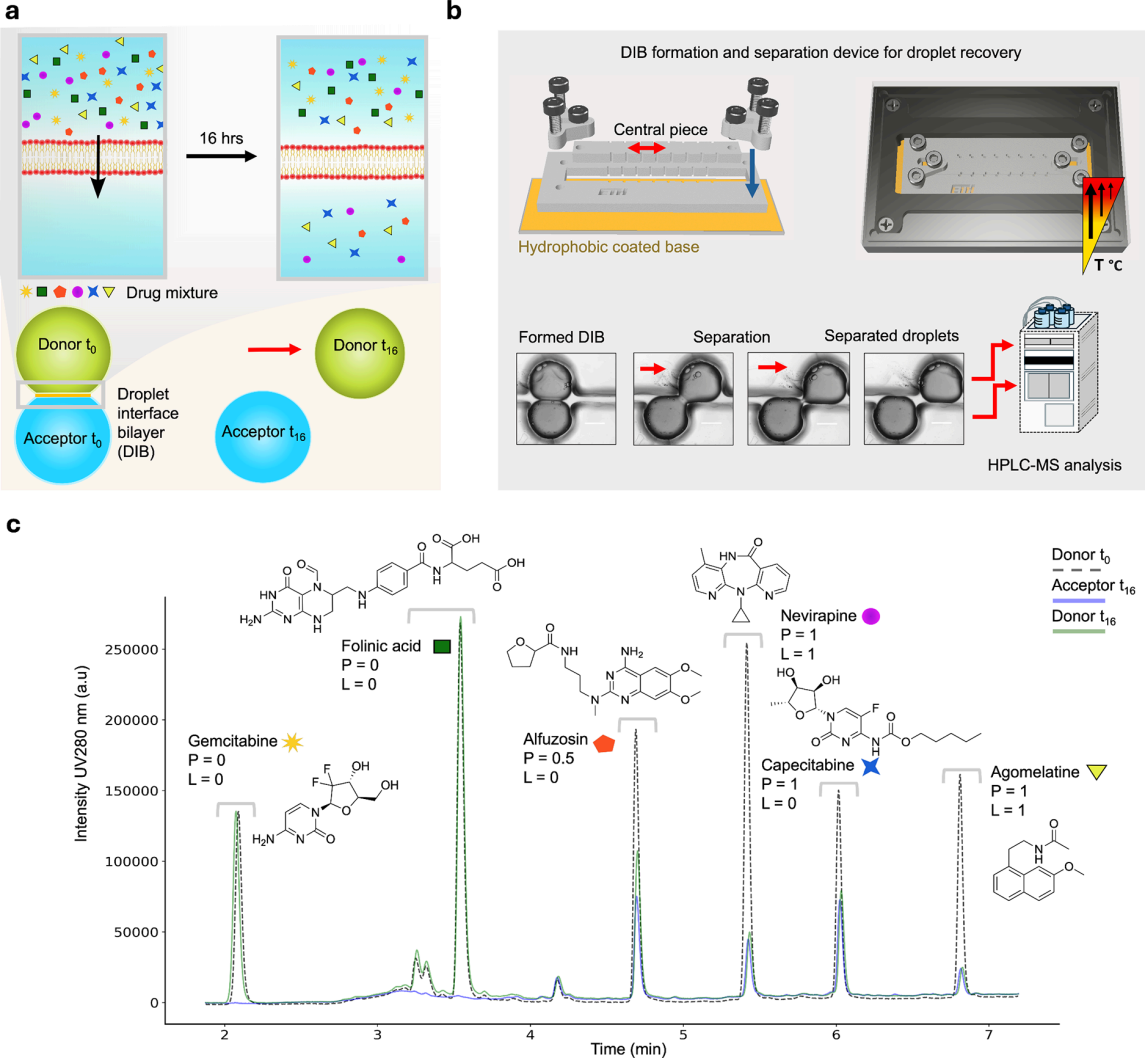
Simultaneous permeability measurements of drug mixtures. (a) Cartoon showcasing the concept of drug mixture analysis. Initially, the donor droplet contains the drug mixture and the acceptor droplet is empty. The mixture of drugs is sorted based on the propensity of each to permeate the interface. Permeable drugs can translocate the interface, and impermeable ones cannot. (b) A heat-controlled open droplet microfluidic system for controlled DIB formation, heating, disconnection, and droplet recovery. Brightfield micrographs show the process whereby DIB lipid monolayers disconnect. Scale bars indicate 0.5 mm. (c) Chromatogram for a drug mixture analysis with six drug peaks. The graph shows three overlapped conditions, the initial concentration of the donor droplet (Donor t_0_), the acceptor droplet after 16 h (Acceptor t_16_), and the donor droplet after 16 h (Donor t_16_). Peak distributions across the three chromatograms qualitatively show the drug’s permeability propensity. Classification values are shown for the permeability classifier (*P*) and the signal loss classifier (*L*) (Supporting Information). Acceptor t_16_ and Donor t_16_ chromatograms are from pooled droplets of at least eight repeats of individual DIBs.

In this study, we carried out a comprehensive analysis of diffusive drug transport across DIBs using a highly parallel approach. Physicochemically diverse mixtures of drugs are placed in one droplet, one side of a DIB. After a defined incubation time, both droplets are disconnected, removed from the device, and measured downstream with high performance liquid chromatography–mass spectrometry (HPLC-MS) to assess the concentration of each target in each droplet. We assayed a library of Food and Drug Administration (FDA) approved drugs and compared our analysis with pharmacological metrics including absorption, distribution, metabolism, and excretion (ADME) predictors. Moreover, we show for the first time heat-enabled reconstitution of bacterial membranes for direct drug-permeation studies and analyze drug permeation in the presence of membrane proteins.

## Results and Discussion

### Platform Design for DIB Production and Disconnection

We designed a method to form biomimetic DIBs and measure multiple drugs label-free (Figure 1a,b). We used a device based on precise droplet placement and indexing, with controlled introduction, simultaneous disconnection, and recovery for downstream HPLC-MS analysis.^36^ The device has four lines, each containing eight droplets resting on a hydrophobic surface. Two lines are on the central piece, and two are on the surrounding chip body. The central piece can be actuated to control the droplet contact. By actuating one of the droplets and fixing the other, a DIB can be split apart by unzipping the contacted lipid monolayers, where droplets can then easily be recovered with a pipet. We modified this platform through inclusion of a bespoke heating chamber (Figure 1b) in which DIBs could reliably be formed after a heating ramp up to an oil temperature of 75 °C (Figure S1). DIB production protocols were similar to those used in previous studies that demonstrated effective sealing against chloride and calcium ions, suggesting relatively defect-free interfaces.^22,30,37,38^

### Assay Design for Drug Mixture Analysis

Next, we developed a label-free assay for assessing drug membrane permeation (Figure 1c). The assay is an end point measurement composed of three distinct chromatograms. The first chromatogram was of the initial composition of the donor droplet. After 16 h, DIBs were disconnected. We chose this time for two reasons; first, that it captured an expected variance in simple diffusion equilibration times observed in the literature and second that it mimicked a reasonable period of oral drug administration, i.e., between doses.^17,30,34,35^ The donor and acceptor were recovered and measured, yielding the two other chromatograms. Each drug could clearly be identified based on the column retention time and where possible, the mass peak. We quantified the propensity for the passive diffusion of each drug via the peak area ratio of the donor and acceptor chromatograms. Each drug was classified as either permeable (*P* = 1, equilibrium reached), slightly permeable (*P* = 0.5 out of equilibrium), or impermeable (*P* = 0, not detected in acceptor, below LOD). The mixture in Figure 1c shows a lipid-only system with at least one drug representing each of the three classifiers and therefore a simultaneous range of simple diffusion rates. This approach also allowed for classification of decay in the sum target signal. Loss may have occurred through a range of mechanisms including solvation into the surrounding oil phase, partition into surrounding lipid micelles,^39^ thermal degradation or precipitation. A similar approach was taken whereby >50% signal loss was classified as high (*L* = 1), 20–50% signal loss was classified as slight (*L* = 0.5) and 20–0% loss was classified as minimal, and the drug was retained (*L* = 0). Further details are given in the Methods section.

### Quantifying Measurable Drug Chemical Space

We first investigated measurable drugs with the HPLC-MS method. To do so, we analyzed a pharmacologically efficacious, structurally and indication diverse, FDA-approved drug library (Figure 2a and Figure S2). Within the library, 70% of drugs were measurable, and the majority were small molecules (<500 Da). We used a hydrophobic column, where retention was defined by exposed hydrophobic groups, and the molecular size. We next screened for hexadecane partition propensity (lipid-free), the oil we formed DIBs in. After incubation in an equal volume hexadecane–water partition, 26 drugs demonstrated >70% decay in signal intensity (Figure 2b) and 26 drugs demonstrated peak splitting (additional peaks or peak fracturing) indicative of degradation. These drugs were considered unsuitable for drug mixture analysis. Within this set there was a clear deviation in calculated octanol–water partition coefficient (log *P*_ow_) distributions indicating that the major driver of signal decay was due to drug hydrophobicity and thus diffusion into the oil (Figure 2c). Peak splitting was distributed at higher molecular weight and polar surface area (Figure 2d,e). Homogenous distributions were observed for other physicochemical metrics (Figure S3). In conclusion, druggable space was analyzable in DIBs below a log *P*_ow_ threshold of 5. The final selection of drugs for the following experiments represented well the initial larger library with respect to physicochemical parameters (Figures S4).

**Figure 2:**
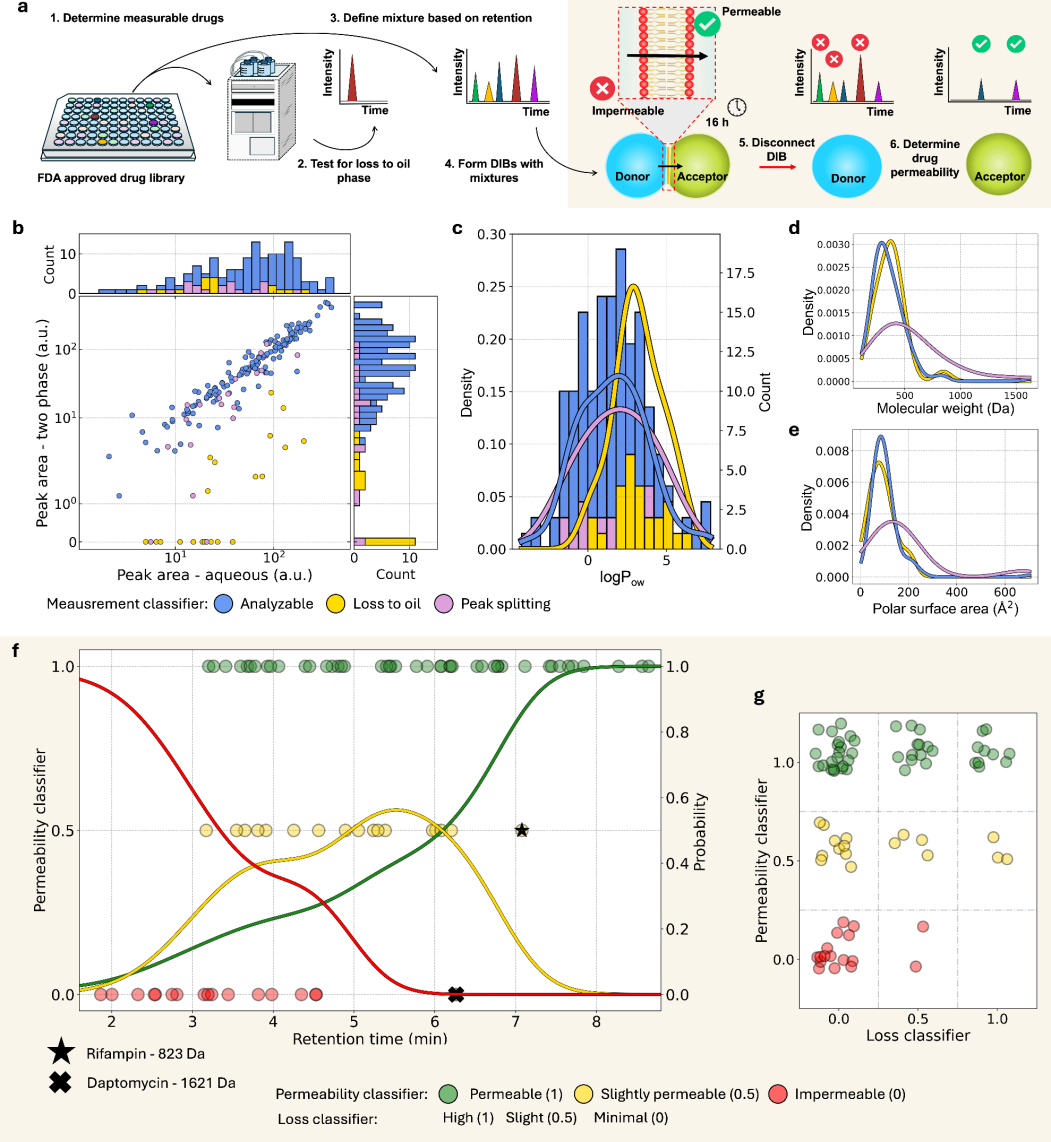
Simple diffusion classification of a drug library. (a) Cartoon of drug mixture analysis applied to a drug library. Workflow indicates relevant assays employed for assessing the suitability of the drug for our method and for classifying the permeability. (b) Loss to the oil phase across the drug library. Central data shows the absolute signal of an aqueous volume containing only the drug (*x* axis) versus a second measurement of the aqueous phase taken after incubation under a water–hexadecane partition (*y* axis). External histograms show peak area distributions per experiment, highlighting the drugs that exhibited total signal decay. Drugs were classified and are color coded according to the measurement classifier. (c–e) Physicochemical properties of the library, visualized as probability density functions of (c) octanol–water partition coefficient (log *P*), (d) molecular weight, and (e) polar surface area. (f) Determined DIB permeability classifiers plotted against the mean retention time (*n* = 4 or 5). Impermeable drugs (red probability distribution) were located at an earlier retention time compared to permeable ones (yellow or green probability distributions). Non small molecule outliers (rifampin and daptomycin >800 Da) were not included in the fitted functions. Lines show probability of classifiers calculated from probability density functions. Data from HPLC-MS. (g) Plotting loss and permeability classifiers for each measured drug. Note that a classification of *P*-0:*L*-1 was not possible with the assay. Data are colored according to the permeability classifier. Experiments were performed with dioleoylphosphatidylcholine (DOPC) bilayers.

### Permeability Classifiers Across Druggable Space

We used the individual retention times to assemble signal-separated mixtures of drugs in the HPLC-MS method. In total, 79 drugs were analyzed in 12 distinct mixtures (Figure 2f, raw data in Note S1). A total of 45 drugs were classified as permeable (*P* = 1), 17 as slightly permeable (*P* = 0.5), and 17 as impermeable (*P* = 0). Permeable small molecule drugs (<800 Da) occupied a retention time range between 3.2–8.65 min. Those classified as impermeable clustered around an earlier, narrower retention range (1.86–4.52 min) and those deemed slightly permeable occupied an intermediary range (3.17–6.2 min). This same relationship could be visualized through a probability distribution generated with kernel density estimation (KDE) (overlaid lines in Figure 2f). At extreme retention times, the probability of permeable or impermeable classifications approached 1. All drugs remained measurable despite the net signal decay observed for 40.5% of drugs (*L* = 0.5 or 1). This may be explained by our permeation experiments introducing new mechanisms for drug concentration loss such as into the oil-micelles as well as an aqueous to oil volume ratio intrinsically biased toward oil partition. The propensity for simple diffusion appeared uncorrelated from this signal decay, as shown by a lack of distinct correlation between the two classifiers (Figure 2g). Diverse permeability and loss classifications were observed across the indications (Figure S5).

### Correlation of Permeability Classifiers with Physicochemical Metrics and ADME Predictors

Many efforts have sought to predict drug-likeness from molecular structure such as the Veber rules and Lipinski’s rule of 5.^40,41^ These commonly applied guidelines originate from correlations between physicochemical properties and appreciable oral bioavailability (rules in Figure 3a caption). Here, we benchmark our results against these rules to investigate their relation to our classifications. The violation of any single rule (Lipinski or Veber) was not absolutely correlated to our permeability classification. As expected, slightly permeable or impermeable drugs were likely to break a rule, but we also found 21 drugs of these categories compliant with the rules (Figure 3a). Of the permeable drugs, 13.5% violated at least one drug-likeness guideline compared to 35.3% and 41.2% of those classified slightly permeable or impermeable, respectively. Each impermeable drug that violated a rule violated the Veber PSA guideline. We further investigated the number of violations (i.e., a drug breaking both MW ≤ 500 and hydrogen bond acceptor groups (HBA) ≤ 5 had two violations). Interestingly, despite the expectation that the number of violations would decrease the likelihood for simple diffusion, coviolations were not exclusive to less permeable drugs (Figure 3b). Increased molecular weight typically coincided with further guideline violations due to the increased surface area and number of functional groups. Importantly, two <500 Da impermeable drugs (pemetrexed and leucovorin) still violated three guidelines.

**Figure 3:**
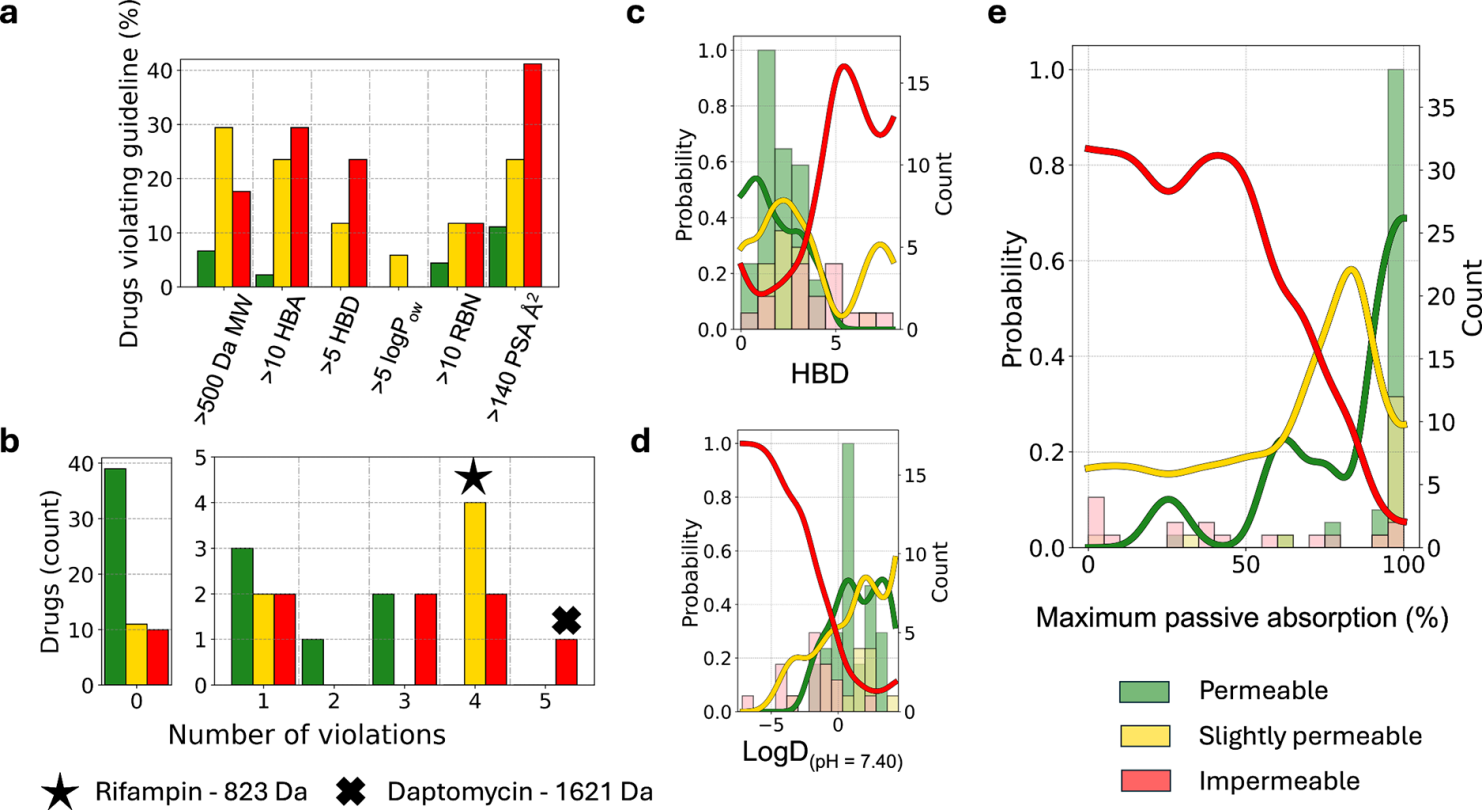
Correlation with physicochemical metrics and a gut absorption predictor. (a) Drug-likeness guideline violations across DIB permeability classifiers. Lipinski guidelines: molecular weight (MW) ≤ 500 Da, number of hydrogen bond acceptor groups (HBA) ≤ 10, number of hydrogen bond donor groups (HBD) ≤ 5, and calculated octanol–water partition coefficient (log *P*_ow_) ≤ 5. Veber guidelines: ≤10 rotational bonds (RBN) and polar surface area (PSA) ≤ 140 Å^2^. (b) Number of guideline violations per drug, per classification. (c–e) Probability functions across calculated ADME metrics and predictors of <800 Da drugs. Data shows KDE fitted histograms. (c) HBD is the clearest differentiator of impermeable (*P* = 0) to permeable (*P* = 1) from the Lipinski guidelines. (d) Distribution coefficient log *D* (pH 7.4), a lipophilicity metric. (e) Maximum passive gut absorption. All metrics and predictors were calculated with ACD/Labs Percepta.

Guideline violations alone were only loosely correlated to the permeability classifiers. Delving deeper, we sought to investigate how our classifications compared to physicochemical metrics computed with available software and current models. As before, probability distributions were generated to visualize the permeability classifications distributed over these metrics. Of the simple drug-likeness metrics, HBD demonstrated a well-separated probability distribution (Figure 3c and d), clearly showing the drugs which violate a Lipinski rule (≤5 HBD). Permeable drugs had a mean and median HBD of 2 aligning with a mean HBD of FDA approved orally dosed drugs of 2.2, which has been conserved since Lipinski’s 1997 publication despite a general trend toward larger sizes and increased lipophilicity.^42^ The important interplay between charge and lipophilicity was accounted for through log *D* (Figure 3d). As shown by the distribution, drugs with log *D* values below 0 were more likely to be classified as impermeable. Statistical testing revealed significant differences between *P* = 1 vs *P* = 0 distributions (Table S7) for the retention time, log *D*, log *P*, HBA, and HBD with no significance for MW, RBN, and PSA. An ensemble learning algorithm trained on a minimal, identified feature set (retention time, log *D*, and HBD) was 77% accurate at assigning *P* = 1 and *P* = 0 classified drugs (Figure S6). Efforts to correlate physicochemical descriptors to the loss classifier were less successful, however high loss drugs were significantly distributed at log *D* values >1 (Figure S7a).

To compare permeability classifiers in DIBs to ADME predictors, we also assessed predicted passive absorption in the gut (Figure 3e) and cellular permeability (Figure S7b). An in vitro maximum passive absorption of >98.5% was satisfied by 84% of the permeable drugs and >93% maximum passive absorption was held by 91% of the population. Impermeable drugs were distributed uniformly across passive absorption from 0% to 100%. High passive absorption also aligned with increased transcellular as opposed to paracellular transport. Similar trends were also observed for correlations to the calculated permeability coefficients for Caco2 and human jejunum epithelial cells (Figure S7b). Highly DIB permeable drugs had a wide range of predicted cellular permeability coefficients, while impermeable drugs were slower, especially for jejunum cells. Interestingly, across all compared parameters, no significant difference was observed between *P* = 1 and *P* = 0.5, only for *P* = 0 and *P* = 1, indicating larger relative structural variation between impermeability and measurable permeability. In tandem, these appreciable correlations to numerous drug absorption parameters show that simple diffusion selectivity across DIBs appears to be pharmacologically relevant. All data from the library analysis is tabulated in Note S1.

### Investigating Passive Transport Mechanisms in Higher Mimetic Systems

Having focused on the drug structure, we went on to investigate biophysical parameters on permeation. Antibiotic efficacy is majorly barriered by the bacterial outer membrane.^43,44^ Mechanisms of resistance can develop around membrane transport.^8^ Cyclic peptides which are classified beyond rule of 5 (bRo5) can undergo distinct transport processes which may overcome developed resistance mechanisms, and challenge an understanding of their activity.^15,44–46^ We therefore investigated the maintenance of the passive diffusion classification of the selected antibiotics.

Using our prior analysis, a physicochemically diverse mixture of six antibiotics was designed such that the mixture contained two antibiotics of each permeability classifier (Figure 4a,b). Rifampin is a macrocycle and was included because it was classified slightly permeable yet it violates four Lipinski guidelines and is thus bRo5 (Figure 3b). In the library analysis, each antibiotic was in a distinct mixture of other drugs and, therefore, had not been mixed before. We used the same analysis protocol, and as expected each antibiotic was baseline separated in the chromatogram and could be assigned. In this new mixture, the permeability classifiers were the same as the prior analysis, indicating that cosolutes had no effect on classification (Figure 4b). The composite flux profile was also revealed by adjusting the incubation time interval (Figure S8).

**Figure 4:**
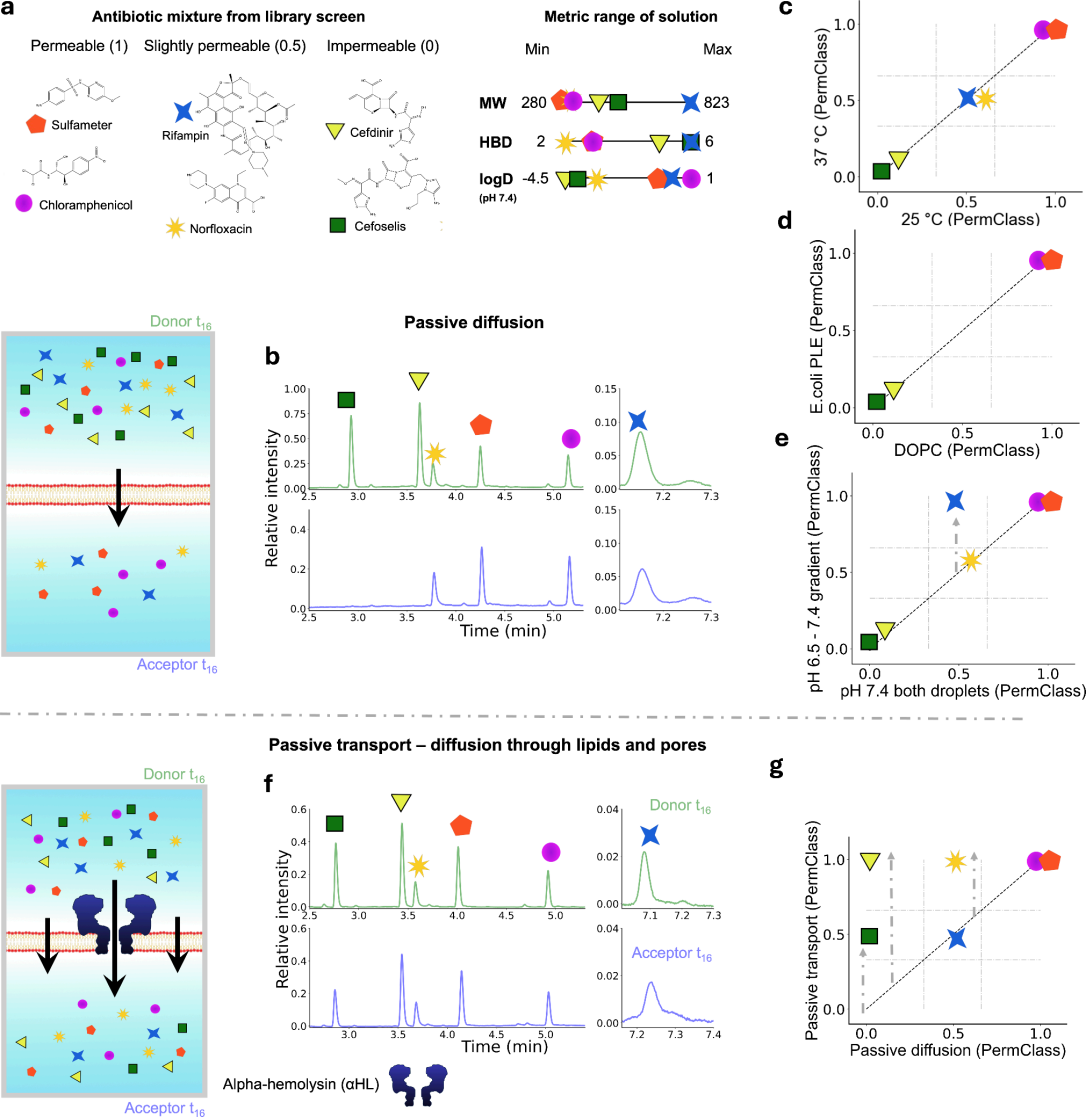
Passive transport mechanism assignment. (a) An antibiotic mixture with varied simple diffusion classifiers. (b) Chromatogram of the acceptor and donor antibiotic mixture after 16 h of incubation at 37 °C. Data from DOPC bilayers. Zoom-ins show rifampin retention region. (c–e) Assessing multiple passive diffusion modulators. (c) Comparing the permeability classifier at 25 and at 37 °C. (d) Comparing a DOPC bilayer and an *E. coli* extract membrane at 37 °C. The heating condition required to form the bacterial membrane degraded rifampin and norfloxacin. (e) Comparing pH symmetry and a pH gradient mimetic of the gut. Performed at 25 °C over DOPC bilayers. Across conditions (c)–(e), all impermeable antibiotics remained so; only rifampin shifted to a permeable classification under the pH gradient. (f,g) Assignment of simultaneous passive transport mechanisms. (f) Chromatogram of the acceptor and donor antibiotic mixture after 16 h of incubation at 37 °C across a DOPC bilayer with a reconstituted protein pore. (g) Comparing the permeability classifier between the data in (b) and (f). Data shows the relative change for each drug via passive transport. Cartoons show distinction between systems reconstituted for passive diffusion and passive transport.

Elevating the temperature of bilayers can increase their permeability.^47^ For DOPC bilayers we found no deviation to any of the six permeability classifiers when elevating to an experimental temperature of 37 °C, indicating the maintenance of structural integrity and lack of a phase transition. Permeation is also tightly linked to the lipid composition of the bilayer. Following a protocol laid out in Taylor et al., we next successfully reconstituted a bacterial lipid bilayer from *Escherichia coli* polar lipid extract.^22^ Compared to a DOPC bilayer, the permeability classifiers held for permeable (1) and impermeable (0) antibiotics. However, due to the requirement of a heating ramp to 50 °C to reconstitute the bilayer, both rifampin and norfloxacin degraded, indicated by no discernible peaks in the chromatogram (Figure S9).

Each of the six antibiotics may be orally administered and, therefore, upon absorption from the intestine to the blood, will transition from an acidic to a basic pH environment. Inspired by a protocol in Korner et al.,^48^ we mimicked the gut pH gradient through modulation of the donor solution to pH 6.5 with the acceptor solution at pH 7.4. In this experiment, rifampin shifted from a permeability classifier of 0.5 to 1 (Figure 4e). If one ion state permeates slower than the other, an ion trapping effect can occur due to the resultant asymmetry in the net flux direction (D to A or A to D) (Figure S10a).^49^ This can aid gut absorption and drug accumulation in lysosomes.^50^ In the pH gradient DIB system, the acceptor peak area was on average 2.19, 1.52, and 1.23 times greater than the donor signal for sulfameter, rifampin, and chloramphenicol, respectively. The preservation of this signal imbalance indicated the maintenance of pH asymmetry across the experimental time frame. This behavior was in line with higher net charged ionic forms at pH 7.4 for all six antibiotics. Despite this, however, the only strong ion trapping effect was for sulfameter, under a shift in charge state from −0.62 to −0.93 ve (Figure S10b–e).

Finally, we applied a drug mixture analysis for passive transport. To add an additional transport mechanism, we formed DIBs in the presence of alpha-hemolysin (αHL), a well understood alpha toxin that forms a beta-barrel pore.^51^ We used a protein unit (960 U/mL) which we previously characterized and assessed in DIBs.^30^ Despite the presence of αHL, droplets could still be separated by using our device. The inclusion of the pore rendered all antibiotics in the mixture membrane permeable (Figure 4f,g) including the simple diffusion impermeable cefoselis and cefdinir. Additional biological repeats gave identical permeability classifiers (Figure S11). These antibiotic measurements align strongly with our previous studies of fluorescent dye transport through αHL. Antibiotic charge was insufficient to explain these observations (Figure S10c). Further modeling revealed a dependency on antibiotic steric and hydration bulk relative to the pore bottleneck of 1.4 nm (Figure S11b).^51^ Cefoselis, the larger of the two previously simple diffusion impermeable antibiotics, was slightly permeable (0.5) through the αHL pore, while cefdinir was permeable (1). Rifampin was classified as slightly permeable in both passive diffusion and passive transport systems, indicating no net change in transport mechanism due to its size. Norfloxacin, smaller than both cefoselis and cefdinir, shifted from slightly permeable to permeable in the passive transport system, indicating transport both through the pore and via simple diffusion.

## Conclusions and Outlook

The chemical and functional diversity of biological membranes has complicated our understanding of how drugs get into biological cells. In this work, we introduce a simple to use method which allows for simultaneous, rapid measurement of druggable chemical space, label-free, under biomimetic conditions. While it is possible that drug–drug interactions may have affected individual translocation rates, our experiments highlight that our simple ternary classification was unaffected by the composition of the drug mixture and is thereby ideal for investigating large net transport rate changes. Owing to the bilayer architecture of DIBs, our assay allowed investigation of facilitated transport alongside simple diffusion, where by exploiting the physicochemical diversity of analytes, we classified the interfacial transport properties. With a selected range of simple diffusion impermeable molecules of varying size, drug mixture analysis may allow for rapid assessment of a reconstituted pore protein,^37,53^ to experimentally ratify protein properties and functionality. Our method is highly scalable, since the number of analytes is limited only by target separation and identification conferred by the HPLC-MS method and thus may be deployed for large libraries at high throughput. Drug mixture analysis may be further applied to model drug coadministration, label-free measurements in complex buffer fluids, measuring ion trapping, and assessing membrane integrity or pore formation. This will prove useful in efforts to reconstitute and study further mechanisms, such as active transport or endocytosis. Increasing the transport complexity has important implications, such as the challenge of maintaining pH asymmetry in the presence of nonspecific pore proteins. Nonetheless, with drug mixture analysis, researchers can extract more and, as we suggest, high quality drug pharmacokinetic information from low sample volumes.

One of the key takeaways from our analysis is that simple diffusion over DIB interfaces is in line with pharmaceutical understanding of gut absorption for MW < 800 Da drugs. We found light correlation to the Lipinski and Veber rules with interesting deviations including permeation of a bRo5 antibiotic. An appreciable rate of simple diffusion was indicated by a log *D* between 0 and 1 and a HBD < 3. These observations underscore the unique engineering opportunities for biomimetic assay and system design with DIBs.^28,54,55^ Comparing such correlations across other planar bilayer architectures will be valuable, and we believe these methods can support the development of models and software to more accurately predict structure–permeability relationships. This analysis therefore strongly supports DIBs as a more biomimetic membrane permeation assay than PAMPA as inferred by previous studies.^16,17,56–58^ However, direct translation of DIB permeation rates to biological membrane permeation rates is currently limited by the lack of inclusion of glycolipids, the relative surface area of bilayer to proteins, and the membrane phase. Importantly, it is anticipated that these variables will attenuate simple diffusion.^20,59,60^ In any case, cell type variation in drug membrane transport depends on protein expression, lipid composition, and the chemical surroundings. It is in the context of improved quantifications of biological membrane transport rates and mechanistic contributions that such in vitro assays will prove especially useful.

## Methods

### Droplet Interface Bilayers for Drug Mixture Analysis

All materials are reported in the Supporting Information, and additional information for each stage is provided. Mixtures were designed based on a library work up and contained 5–8 drugs depending on the expected peak separation. For DIB formation, we modified previously published protocols for stable bilayers (see Supporting Information). The donor solution for the analysis contained each drug in an equimolar mixture at approximately 50–100 μM (depending on number of cosolutes) with 10 mg/mL, 100 nm DOPC vesicles in DPBS, pH 7.4, 5 v/v% DMSO. The acceptor solution comprised the same solution without drugs.

We previously reported a protocol for device fabrication for droplet connection and disconnection.^28^ We first pipetted 500 nL droplets of donor and acceptor solution into a fabricated device with the donor and acceptor initially separated. Unless otherwise stated, droplet monolayers were stabilized with 1 mg/mL DOPC in hexadecane lipid oil deposited over the top of the device and incubated for 10 min and then introduced to form a DIB. The device was sealed to the surroundings using a lid, and the system was left for 16 h. Five microliters of the donor solution was kept in a fridge overnight. DIBs were simultaneously separated by carefully actuating the device. Separated droplets were then individually recovered using a pipet where only the aqueous phase was pooled into a HPLC vial. At least eight individual droplets of the same experimental identity were pooled per measured sample, to facilitate sufficient volume for a stable HPLC-MS injection of 1 μL. All samples were analyzed via a previously published HPLC-MS method (see Supporting Information).

### Antibiotic Mixture Analysis

As above, the donor solution contained ca. 100 μM each purchased antibiotic, 5 v/v% DMS0 in a 10 mg/mL 100 nm DPBS vesicle solution. The acceptor solution had the same composition, barring the antibiotics. For bacterial mimetic DIBs, droplets were first incubated at 50 °C for 1 h, allowing *E. coli* lipid extract monolayers to form.^22^ We then introduced droplets at 50 °C, where they formed a DIB. The system was then cooled to 37 °C and kept there for the following 16 h after the same experimental protocol for permeation analysis was run.

### Data Analysis

All peak fitting was performed with ChemStation OpenLAB software from Agilent. We interpreted and reported the peak areas for our experiments. If HPLC-UV analysis was not feasible, then we interpreted the MSD peak and area. Drugs were assigned classifiers according to the peak area ratio between the donor and acceptor for permeability and the sum area values for loss. All physicochemical and ADME data were generated with the Percepta software from ACD/Labs (Release 2024.1.0), licensed by the Chemistry | Biology | Pharmacy Information Center, ETH Zurich. Additional data analysis was carried out with Python to determine probability distributions and conduct statistical testing.

## Supplementary Material



## Notes

Extended methodology, tabulated data, and additional analysis (PDF)
