# Supplementary material for: Passive Membrane Transport Analysis of Drug Mixtures
Source: Anal Chem. 2025 Aug 5;97(32):17472–80. doi: 10.1021/acs.analchem.5c02264 (PMC12368836; doi:10.1021/acs.analchem.5c02264)
Supplement: Supplementary file 1 [file ac5c02264_si_001.pdf]

Supporting Information

Passive membrane transport analysis of drug mixtures

Robert Strutt[1], Simon F. Berlanda[1], Petra S. Dittrich[1]*

1 - Department of Biosystems Science and Engineering, ETH Zürich, Schanzenstrasse 44, 4056 Basel, Switzerland

* = corresponding author – email: petra.dittrich@bsse.ethz.ch

Table of contents

Item	Page
Supporting materials and methods	S2 - 5
Figure S1: Device and heating characterisation.	S6
Figure S2: Library indication space.	S7
Figure S3: Additional physicochemical analysis of measurement classifications.	S8
Figure S4: Permeability measurements captured the broad, representative physicochemical space of measurable drugs.	S9
Note S1 (Tables S1 – S6): Tabulated data	S10 - 20
Table S1: Lipinski and Veber guideline metrics for analyzed drugs.	S10 - 11
Table S2: Brain transport and brain transport metrics for analyzed drugs.	S12 - 13
Table S3: ADME predictors of intestinal absorption and bioavailability for analyzed drugs.	S14 – 15
Table S4: Bulk loss analysis for analyzed drugs.	S16 – 17
Supplementary table 5: Drug mixture analysis for measured drugs.	S18 – 19
Table S6: Retention times for measured drugs.	S20
Figure S5: Indication space of classifiers.	S21
Table S7: Statistical testing	S22
Figure S6: Extreme gradient boosting (XGBOOST) assessment of permeability classifiers.	S23
Figure S7: ADME predictors compared probabilistically to loss and permeability classifications.	S24
Figure S8: Mixture flux profile	S25
Figure S9: Antibiotic mixture analysis of passive diffusion across a reconstituted *E.coli* polar lipid extract bilayer.	S26
Figure S10: Ion trapping and pKa assessment of antibiotics exposed to pH gradient.	S27
Figure S11: Additional biological repeat of passive transport experiment.	S28
References	S29

Supporting materials and methods.

Materials

Dioleoyl phosphatidylcholine (DOPC) and *E.coli* polar lipid extract (both from Avanti Polar Lipids), hexadecane (H6703), DPBS 1X pH 7.4, DMSO for molecular biology (D8418), glass slides (1*75 * 25 mm - S8902), rifampin (PHR180), sulfameter (S0383), norfloxacin (N9890), chloramphenicol (3166), α-hemolysin from *Staphylococcus aureus* (lyophilized powder), were all supplied from Merck. Polydimethylsiloxane (PDMS - SYLGARD^{TM} 184 Silicone elastomer kit) was purchased from DOW Inc. Cefoselis sulfate (B1906) was purchased from LubioScience. Cefdinir in DMSO stock (10mM S1605) was purchased from Selleck Chemicals. The small molecule library (FDA approved drug collection (Selleck Chemicals)) was purchased from NEXUS Personalized Health Technologies. Temperature was recorded using a Thorlabs TSP01 USB temperature and humidity data logger. Polymethylmethacrylate (PMMA - 2 mm transparent) was supplied by Sheet Plastics UK. All HPLC measurements were performed in 1.5 mL screw neck vials (080401-W) with 150 µL conical glass inserts (110500) and sealed with Septa silicone / PTFE caps (070301), all supplied from BGB Analytik. All HPLC-MS was performed with LC-MS grade reagents.

HPLC-MS method

In line with our former protocol we used an Agilent 1260 II Infinity LC attached to an atmospheric pressure ionization-electrospray (API-ES) single quadrupole, Agilent G6130B mass spectrometer.[1] Here, the mass selective detector (MSD), was run for all experiments, simultaneously analyzing with the LC at 280 nm and an m/z scan range of 50 – 1000 in the MSD. For the continuous phase, we used a linear 99.5: 0.5 acetonitrile:formic acid and LC-MS grade water gradient. The column was a reversed-phase Poroshell 120 C18 2.17 µm 3.0x150 mm column set at 60 °C. Where possible, all peaks were assigned based on identification of the target molecular weight in the mass spectrum determined with the MSD and marked in the tables of Note S1.

Library work up

Each well of the library was supplied with a DMSO stock of each drug (10 mM). The library was worked up in batches using a Dispendix I.Dot acoustic liquid handler, to co-dissolve the DMSO with DPBS 1X buffer in selected rows of the library plate. To initially determine the fraction of detectable drugs, a sample of each well was then further diluted into a DPBS buffer (v/v%) at approximately 200 µM, 5 µL. To minimize evaporation of the plate between runs, a sealing film was applied to the library, and it was kept in a -20 °C freezer. We measured 1 µL of this aqueous sample with the HPLC-MS to obtain the x axis values in Figure 2 b. To eliminate drugs with high oil partition, we next added 4 µL of hexadecane oil on top of the previously measured aqueous sample. We left the sample at room temperature for 16 h and the sample was then reanalyzed, ensuring that the injection needle passed through the interface and withdrew 1 µL again from the aqueous layer. This second value formed the y axis in Figure 2 b. Peak splitting was defined by additional peaks appearing in the second run, typically with a significant signal decay in the same peak from the initial experiment. Drugs were classified as 'Loss to oil' if they demonstrated >75% signal loss in peak area between runs. Of the 299 assessed drugs, 208 yielded measurable, discernible peaks. Of the 91 immeasurable, 32 were deemed likely insoluble (an HPLC peak was expected due to significant aromaticity) and 59 had no expected peak. Both measurement behaviors were not suitable for our DIB analysis.

DIB device fabrication

Chips were laser cut from transparent 2 mm thick PMMA in a Universal Systems VLS 2.30 CO_2 laser cutter with a high-power density focusing optics (HPDFO) lens. Our device held 32 droplets and thereby 16 DIBs. In this work, we used a custom heating chamber. This chamber featured two heating elements along its width. The chamber was sealed to hexadecane leakage and the bath (ca. 8 mL total) could be heated using a connected proportional integral derivative (PID) controller. The temperature of the oil was tracked through insertion of the temperature probe. The offset for system heat loss was accounted for by setting the PID. All experiments report the measured oil temperature. We produced PDMS coated glass slides using the previously published protocol. The entire device fit atop a VWR backlit stereo microscope which was used for droplet indexing, visualizing DIBs and droplet recovery.

Lipid preparation
Lipid films were formed by evaporating away chloroform from lipid stocks in a disposable 8 mL cylindrical glass vial. Films were left for at least 12 h in a desiccator to remove lingering chloroform. A lipid vesicle suspension was formed by dissolving the film in DPBS, followed by extrusion through a 100 nm pore polycarbonate filter at least 20 times using an Avanti extruder set. Lipid oil was formed by dissolving the film in hexadecane and sonicating the solution at 37 °C for 1 h.

Drug mixture analysis
To maintain diverse chemical structures within each mixture, compositions were chosen based on expected peak separation and varied co-ions (i.e. no mixtures with 5 HCL partnering ions). Care was taken to ensure that all mixtures never exceeded a DMSO v/v% of 5%. At these ranges, DMSO has previously been shown to be negligible on the rate of simple diffusion.[2] The broad library analysis was performed at 25 °C across DOPC lipid bilayers. Each mixture was assigned a reference number, for example, the data in figure 1 shows data from mixture 9. Working from the library was a 'one shot' assay with a <20 μL donor volume per mixture. Subsequent droplet additions into the vial led to droplet coalescence and an approximal analyte volume of at least 4 μL (8*500 nL droplets) per side of the assembled DIB system. Throughout the library analysis, we always had at least 8 recoverable droplets per condition. This ensured a high number of statistical repeats and fulfilled sufficient volume for analysis of each experiment. In one instance (mixture 4), the acceptor solution had an air bubble, which led to a compromised solution intensity. To facilitate quantification, we repurchased these drugs individually and performed the analysis again, following the same conditions. We report and interpret this data for the manuscript. Qualitatively, the peak response, i.e. impermeable or permeable for each drug was identical between the experiments.

Antibiotic mixture analysis
Of drugs that violated a guideline (Figure 3 a), 33.3% were antibiotics of a representative 12.7 % in the overall analyzed population. We prepped DMSO stocks of each purchased antibiotic, which were then co-dissolved into DPBS stocks. These stocks were then mixed at an equal molar ratio. For bacterial mimetic DIBs, all *E.coli* PLE films were freeze-thawed 3 times before extrusion. For these experiments we used a lipid in (lipid only in the aqueous phase) exclusive approach, to satisfy a mixture representative monolayer coverage. Bilayer formation was observed through preclusion of the inter-droplet oil layer. For pH gradient experiments, the donor solution was modified from pH 7.4 to 6.5 and the same protocol was run. We noted increased DIB instability for this experiment. Experiments with αHL were lipid out only (lipids only in the oil phase), the donor and acceptor solutions were the same, however, the acceptor solution additionally contained 960 U/mL αHL. αHL inserted into the bilayer from the opposite direction of the mixture concentration gradient. Descriptions of the unit can be found on the suppliers website. It was previously shown that the insertion direction relative to a chemical gradient is negligible on the reconstitution efficiency.

Additionally in the same study, the tested concentration was shown to form stable membranes.[3]

Classifier allocation

The permeability classifier (P) took the peak area ratio between the donor ($I_{Dt=16}$) and acceptor ($I_{At=16}$) droplets after 16 h.

$$I_{ratio} = \frac{I_{At=16}}{I_{Dt=16}} \quad (1)$$

Where:
$I_{At=16}$ = The integrated peak area of the drug in the acceptor droplet (A) after 16 h incubation.
$I_{Dt=16}$ = The integrated peak area of the drug in the donor droplet (D) after 16 h incubation.
I_{ratio} = The integrated peak area ratio comparing the relative peak magnitude.

I_{ratio} > 0.85 → P = 1 ≡ Permeable, assigned to 45 drugs.
I_{ratio} < 0.85 and $I_{At=16}$ < LOQ → P = 0.5 ≡ Slightly permeable, assigned to 17 drugs.
$I_{At=16}$ < LOQ → P = 0 ≡ Impermeable, assigned to 17 drugs.

Drugs with a high passive diffusion propensity and therefore fast translocation rates were classified as P = 1. Within 16 h, these drugs had reached chemical equilibrium across both droplets, as indicated by peak area overlap (± 15% tolerance >0.85 peak ratio). For slow diffusing drugs, which after the period had not reached chemical equilibrium, these were classified as P = 0.5. The long timeframe facilitated enough concentration of the drug to accumulate in the acceptor droplet (as depleted from the donor) and enable peak quantification (approximately 1uM). For drugs that were undetectable in the acceptor droplet after 16 h, these were classified as P = 0. In this work, we refer to drugs with this classification as 'impermeable'.

The loss classifier (L) was based on the sum of acceptor ($I_{At=16}$) and donor ($I_{Dt=16}$) peak area as a percentage of the initial donor ($I_{Dt=0}$) peak area:

$$I_{loss} = 100 - \left(\frac{I_{At=16} + I_{Dt=16}}{I_{Dt=0}} * 100\right) \quad (2)$$

Where:
$I_{Dt=0}$ = The integrated peak area of the drug in the donor (D) droplet prior to incubation at t=0.
I_{loss} = The % signal lost from the point of droplet formation to the point of droplet recovery after DIB formation.

I_{loss} > 50% → L =1 ≡ High loss.
I_{loss} – 20– 50% → L = 0.5 ≡ Slight loss.
I_{loss} – 0 – 20% → L = 0 ≡ Minimal loss.

Physicochemical property generation and ADME predictors

Structures were calculated from the SMILES strings supplied with the library. The organic part of the molecule was calculated by the software in the case of salts and co-ions. Unless stated we used a pH of 7.4, a spin of 500 rpm for simulating mixing conditions and a dosage of 10 mg for bioavailability calculations. Drug likeness violations - two permeable drugs had three guideline violations (etoposide and darunavir), four slightly permeable drugs had four guideline violations (doxorubicin, olmesartan, adefovir dipivoxil and rifampin) and five impermeable drugs had three or more guideline violations (pemetrexed, leucovorin, cefditoren pivoxil, cefoselis sulfate and daptomycin). The KDE analysis was undertaken with SciPy gaussian_kde function using the 'scott' bandwidth selection method. Correlation in

ADME predictors to DIB permeability classifiers were marked by regions in the probability distribution with cumulatively high probabilities (>0.7) to be classified either permeable (P = 1 or 0.5) or impermeable (0). The distributions additionally provide insight on the relative spread within classifications (multiple figures throughout). Statistical testing was performed using a Kruskal-Wallis test for assessing if at least one of the 3 distributions were different from another. We then used a pairwise Mann-Whitney test for each pair of distributions to test significance between under assumption of non-normal distributions, as observed. All metrics are included and further detailed herein.

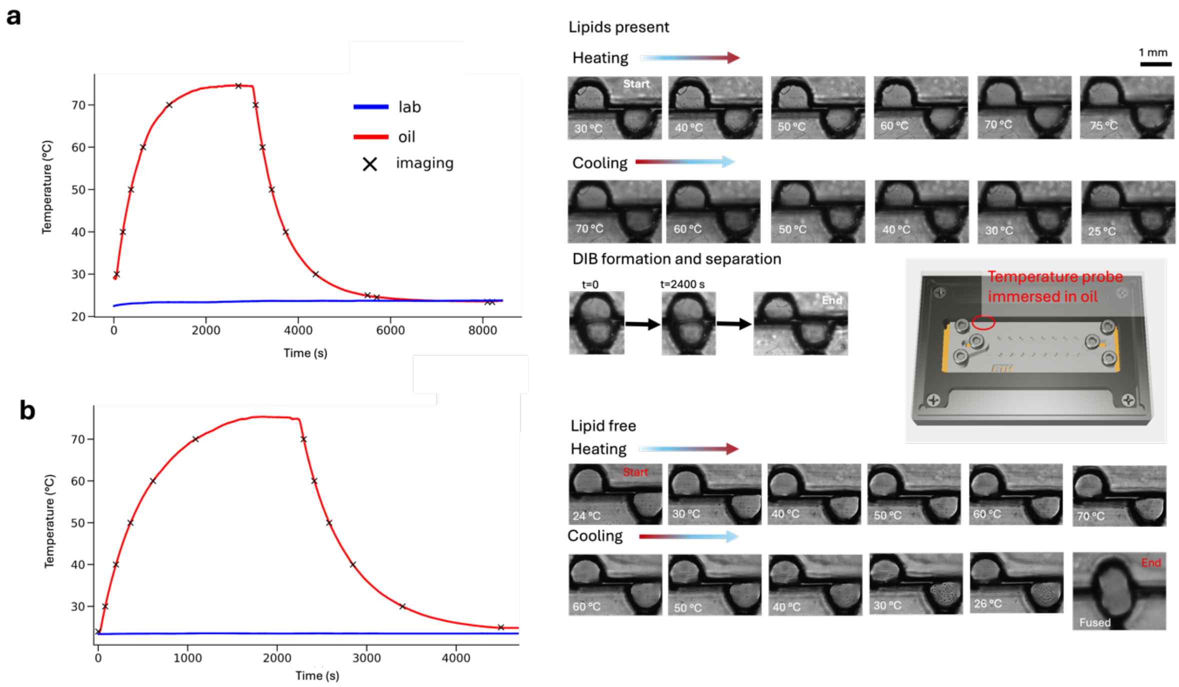

Figure S1: Device and heating characterisation. (a) DIB formation after heat ramp, here shown with DOPC dissolved in the oil phase (2 mg/mL). Temperature heat ramp recorded in oil and surrounding temperature. Measurements were recorded with a temperature probe. A recording probe was inserted into the oil phase of the device. DIBs could be reliably formed and separated after a heat run, up to temperatures of 75 °C **(b)** Without lipids, droplets coalesced after a similar heat run. Droplets displayed no visible evaporation within experimental timeframe. Left hand side shows recorded temperature ramp. Note that the majority of permeation experiments were performed at 25 °C, and we later used this system to reconstitute complex lipid bilayers (see Figures 4 and Figure S8, 10).[4]

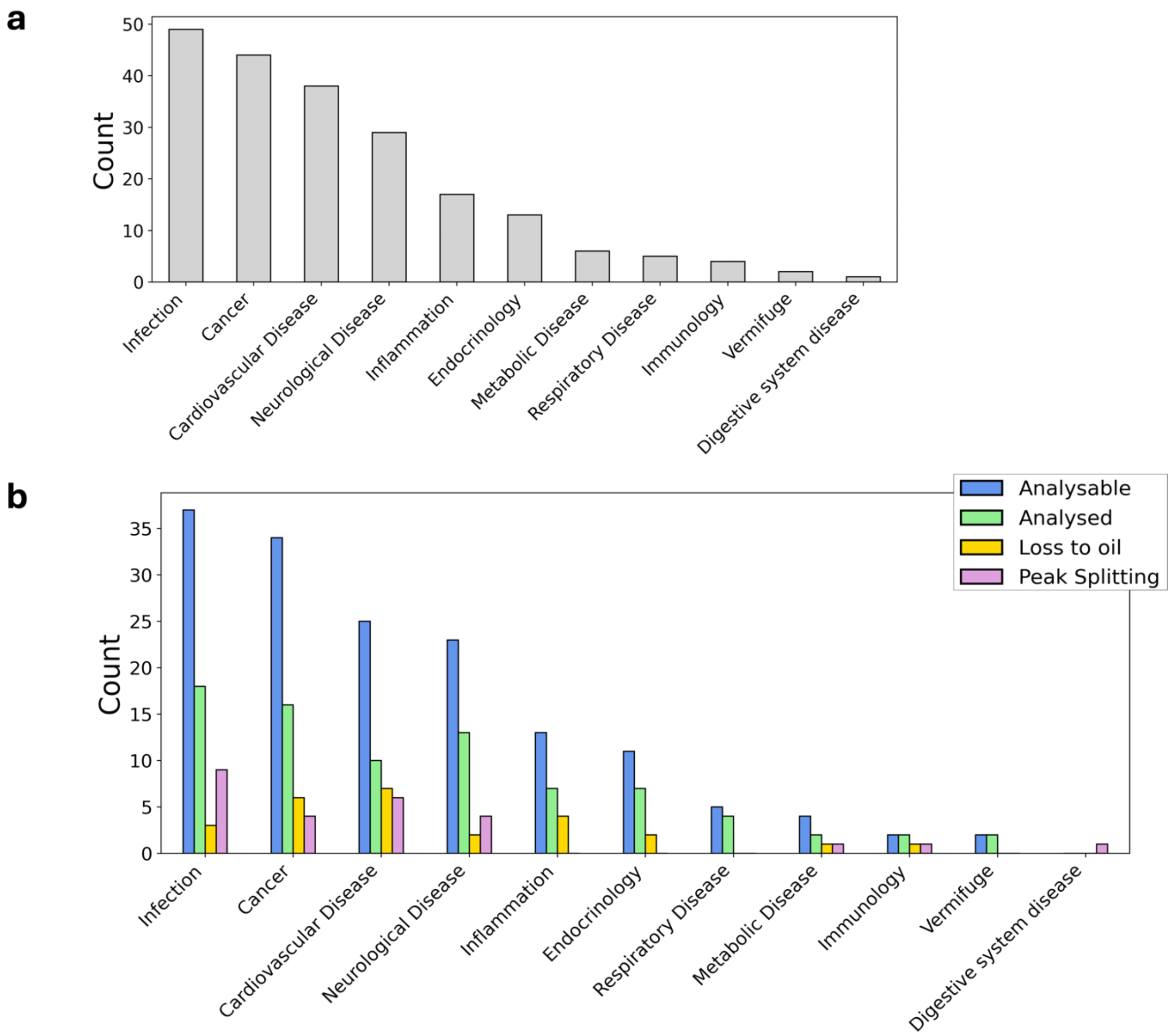

Figure S2: Library indication space. (a) Indication space of all 208 measurable drugs in the library. Indication data supplied with NEXUS chemical library. **(b)** Indication space of measurement classifications.

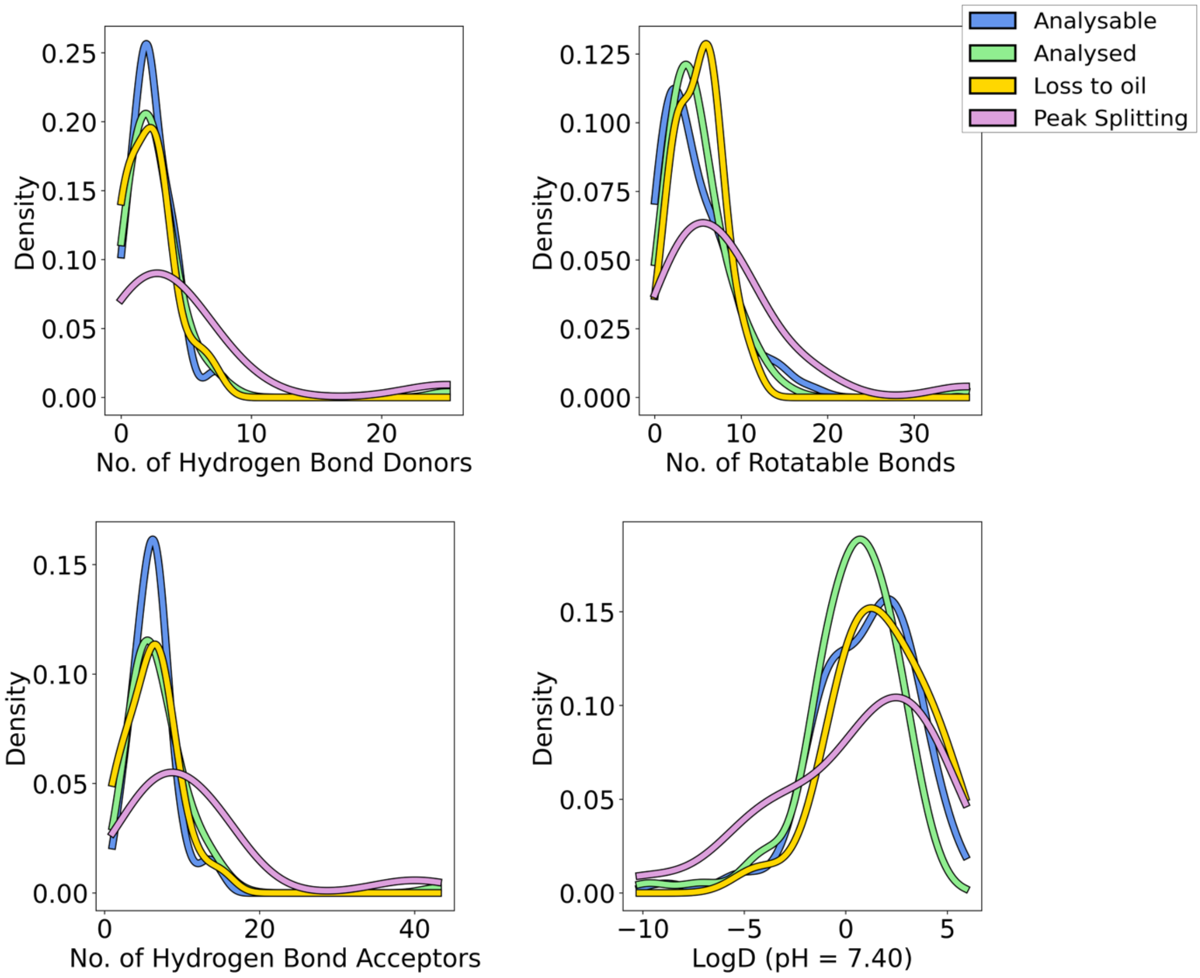

Figure S3: Additional physicochemical analysis of measurement classifications. Significant deviations were only observed for the peak splitting distributions in no. rotatable bonds and no. hydrogen bond acceptors. Both variables are also correlated with increased molecular size (as shown in Figure 2 d and e). All other distributions were highly comparable, indicating that a broad chemical space was analyzable within the system.

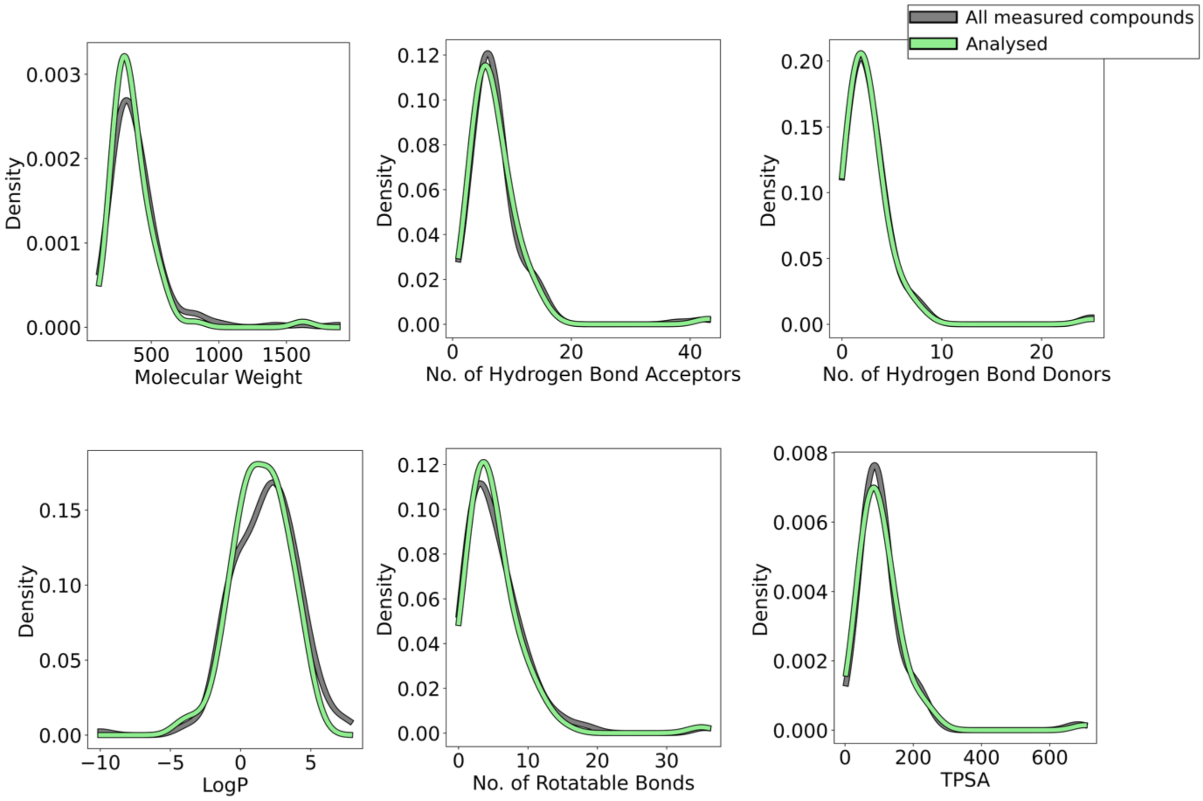

Figure S4: Permeability measurements captured the broad, representative physicochemical space of measurable drugs. Data shows overlap in physicochemical properties between all measured drugs and the drug dataset measured for passive diffusion in DIBs. Data show probability density functions which strongly overlap for all Lipinski and Veber durg-likeness guidelines. The data measured in DIBs therefore captured a physicochemical distribution broadly representative of the larger library.

Note S1: Tabulated data

Here, we have tabulated all data from the drugs analysed with drug mixture analysis. The data has been broken up into sections for readability / use.

Table S1: Lipinski and Veber guideline metrics for analyzed drugs.

Name – The drug name – supplied with the library.
Indication – The indication of the drug – supplied with the library.
MW – Molecular weight measured in Da - calculated with Percepta.
HBD – The no. hydrogen bond donor groups - calculated with Percepta.
HBA – The no. hydrogen bond acceptor groups - calculated with Percepta.
$LogP_{ow}$ – The calculated octanol water partition coefficient - calculated with Percepta.
RBN – The no. rotatable bonds in the molecule - calculated with Percepta.
TPSA – The topological polar surface area measured in Å^2 - calculated with Percepta.

Name	Indication	MW	HBD	HBA	$LogP_{ow}$	RBN	TPSA
Topiramate	Neurological Disease	339.36	2	9	2.15	3	123.92
Ifosfamide	Cancer	261.08	1	4	0.68	5	51.38
Rufinamide (Banzel)	Neurological Disease	238.2	2	5	0.42	3	73.8
Fluoxetine HCl	Neurological Disease	309.33	1	2	4.23	7	21.26
Ipratropium bromide	Respiratory Disease	332.46	1	4	-1.62	6	46.53
Ubenimex (Bestatin)	Cancer	308.38	5	6	1.64	8	112.65
Fluconazole	Infection	306.28	1	7	0.7	5	81.65
Anastrozole	Endocrinology	293.37	0	5	2.67	6	78.29
Saxagliptin	Infection	315.42	3	5	0.71	3	90.35
Pemetrexed	Cancer	427.42	7	11	0.25	9	186.97
Doxorubicin (Adriamycin)	Cancer	543.53	7	12	0.92	5	206.07
Leflunomide	Inflammation	270.21	1	4	2.2	3	55.13
Repaglinide	Endocrinology	452.6	2	6	4.88	10	78.87
Ofloxacin (Floxin)	Cancer	361.37	1	7	0.17	2	73.32
Chloramphenicol (Chloromycetin)	Infection	323.13	3	7	0.86	6	115.38
Dyphylline (Dilor)	Respiratory Disease	254.25	2	8	-0.94	3	98.9
Olmesartan medoxomil (Benicar)	Cardiovascular Disease	558.6	2	12	4.09	11	154.34
Nevirapine (Viramune)	Infection	266.3	1	5	2.1	1	58.12
Cefditoren pivoxil	Infection	620.71	3	13	2.46	12	257.18
Etodolac (Lodine)	Inflammation	287.36	2	4	3.42	4	62.32
Dorzolamide HCL	Cancer	324.43	3	6	-0.22	3	151.33
Ramelteon	Neurological Disease	259.35	1	3	2.8	4	38.33
Marbofloxacin	Infection	362.36	1	8	0.01	2	76.56
Flurbiprofen (Ansaid)	Inflammation	244.27	1	2	3.82	3	37.3
Gemcitabine (Gemzar)	Metabolic Disease	263.2	4	7	-1.36	2	108.38
Cefdinir (Omnicef)	Infection	395.41	5	10	-0.48	5	211.75
Pitavastatin calcium (Livalo)	Cardiovascular Disease	421.47	3	5	3.5	8	90.65
Irinotecan	Cancer	586.69	1	10	3.77	5	112.51
Glipizide (Glucotrol)	Endocrinology	445.54	3	9	1.82	7	138.53
Crizotinib	Cancer	450.34	3	6	4.54	5	77.99
Methylprednisolone	Immunology	374.48	3	5	1.97	2	94.83
Drospirenone	Endocrinology	366.5	0	3	3.47	0	43.37
Lamivudine (Epivir)	Infection	229.25	3	6	-0.95	2	113.45
Sulfapyridine (Dagenan)	Infection	249.29	3	5	0.46	3	93.46
Vismodegib	Cancer	421.29	1	5	3.13	4	84.51
Bendamustine HCL	Cancer	358.26	1	5	2.86	9	58.36
Ruxolitinib	Cancer	306.37	1	6	2.31	5	83.18
Leucovorin Calcium	Metabolic Disease	473.45	8	14	-2.41	9	215.55
Alfuzosin hydrochloride (Uroxatral)	Cardiovascular Disease	389.46	3	9	1.02	8	111.83
Sumatriptan succinate	Neurological Disease	295.4	2	5	1.32	6	73.58
Adefovir Dipivoxil (Preveon, Hepsera)	Infection	501.48	2	13	2.2	15	176.79
Pyridostigmine Bromide (Mestinon)	Cardiovascular Disease	181.22	0	4	-3.34	2	33.42
Gemfibrozil (Lopid)	Cardiovascular Disease	250.34	1	3	4.19	6	46.53
Sulfameter (Bayrena)	Infection	280.3	3	7	0.36	4	115.58
Nitazoxanide (Alinia, Annita)	Vermifuge	307.28	1	8	2.06	5	142.35
Ketorolac (Toradol)	Neurological Disease	255.27	1	4	2.58	3	59.3
Floxuridine	Cancer	246.19	3	7	-1.32	2	99.1
Dexamethasone	Inflammation	392.47	3	5	1.92	2	94.83
Flumazenil	Neurological Disease	303.29	0	6	1	3	64.43
Nelarabine (Arranon)	Cancer	297.27	5	10	-0.98	3	148.77

Etoposide (VP-16)	Cancer	588.56	3	13	0.66	5	160.83
Nepafenac	Inflammation	254.29	4	4	1.38	4	86.18
Tianeptine sodium	Neurological Disease	436.95	2	6	3.21	8	95.09
Aminoglutethimide (Cytadren)	Endocrinology	232.28	3	4	1.27	2	72.19
Norfloxacin (Norxacin)	Infection	319.34	2	6	-0.56	3	72.88
Apixaban	Cardiovascular Disease	459.51	2	9	0.87	5	110.76
Thiabendazole	Vermifuge	201.25	1	3	2.39	1	69.81
Amprenavir (Agenerase)	Infection	505.63	4	9	3.22	12	139.57
Adenosine (Adenocard)	Cardiovascular Disease	267.25	5	9	-1.05	2	139.54
Ftorafur	Cancer	200.17	1	5	-0.4	1	58.64
Capecitabine (Xeloda)	Cancer	359.35	3	9	0.61	7	120.69
Cefoselis sulfate	Infection	522.56	6	14	-0.64	9	251.31
Evista (Raloxifene Hydrochloride)	Endocrinology	473.59	2	5	5.48	7	98.24
Ondansetron hydrochloride (Zofran)	Infection	293.37	0	4	2.79	2	39.82
Prazosin HCl	Cardiovascular Disease	383.41	2	9	1.16	4	106.95
Tizanidine HCl	Neurological Disease	253.71	2	5	1.29	2	90.44
Aminophylline (Truphylline)	Respiratory Disease	180.17	1	6	0.13	0	69.3
Azathioprine (Azasan, Imuran)	Immunology	277.26	1	9	0.01	3	143.4
Darunavir	Infection	547.67	4	10	3.26	12	148.8
Guaifenesin (Guaiphenesin)	Respiratory Disease	198.22	2	4	0.84	5	58.92
Daptomycin	Infection	1620.69	25	43	-4.22	35	702.02
Agomelatine	Neurological Disease	243.31	1	3	3.02	4	38.33
Indomethacin (Indocid, Indocin)	Inflammation	357.79	1	5	4.01	4	68.53
Chlorothiazide	Cardiovascular Disease	295.71	3	7	-0.05	1	135.45
Zolmitriptan (Zomig)	Neurological Disease	287.36	2	5	2.37	5	57.36
Piroxicam (Feldene)	Inflammation	331.35	2	7	2.22	2	107.98
Hydrochlorothiazide	Cardiovascular Disease	297.73	4	7	0.01	1	135.12
Deferasirox	Endocrinology	373.37	3	7	4.27	4	108.47
Rifampin	Infection	822.95	6	16	2.22	5	220.15

Table S2: Brain transport and brain transport metrics for analyzed drugs.

Name – The drug name – supplied with the library.
LogBB – The indication of the drug – supplied with the library.
Log(PS*fu, brain) – Brain/plasma equilibration rate. Determined from LogPS – the rate of passive diffusion and the steady state ratio of equilibration across brain tissue and plasma - calculated with Percepta.
Indicated Carrier Transport – Binary indicator if linked directly to carrier transport. Determined from Percepta database.
Indicated Protein Substrate – If 'Indicated Carrier Transport' = 1, this column names the linked protein substrate - determined with Percepta.
Efflux probability – The probability of being a substrate for P-glycoprotein (P-gp) - calculated with Percepta.
Efflux probability RI – The reliability of the probability determination of P-gp efflux - calculated with Percepta.
Indicated Significant Efflux Transport – Binary indicator of significant efflux transport - calculated with Percepta.

Name	LogBB	Log(PS*fu, brain)	Indicated Carrier Transport	Indicated Protein Substrate	Efflux probability	Efflux probability RI	Indicated Significant Efflux Transport
Topiramate	0.31	-2.81	0		0.67	0.54	0
Ifosfamide	0.17	-2.3	0		0.06	0.95	0
Rufinamide (Banzel)	-0.21	-2.64	0		0.3	0.21	0
Fluoxetine HCl	0.62	-3.08	1	Organic cation	0.36	0.61	0
Ipratropium bromide	-0.05	-4.21	0		0.9	0.43	1
Ubenimex (Bestatin)	-0.12	-4.2	0		0.27	0.54	0
Fluconazole	0.05	-2.6	0		0.15	0.48	0
Anastrozole	0.38	-2.27	0		0.26	0.11	0
Saxagliptin	-0.45	-3.45	0		0.43	0.23	0
Pemetrexed	-2	-5.82	1	Nutrient trasnporters	0.03	0.45	0
Doxorubicin (Adriamycin)	-0.65	-4.98	0		0.95	0.95	1
Leflunomide	-0.46	-2.2	0		0.28	0.28	0
Repaglinide	-0.01	-3.92	0		0.49	0.33	0
Ofloxacin (Floxin)	-0.1	-3.65	0		0.14	0.78	0
Chloramphenicol (Chloromycetin)	-0.08	-2.95	0		0.09	0.51	0
Dyphylline (Dilor)	-0.03	-3.52	0		0.07	0.48	0
Olmesartan medoxomil (Benicar)	-0.26	-4.31	0		0.8	0.41	1
Nevirapine (Viramune)	-0.01	-2.28	0		0.2	0.62	0
Cefditoren pivoxil	0.1	-3.48	0		0.56	0.23	0
Etodolac (Lodine)	-0.43	-3.37	0		0.24	0.21	0
Dorzolamide HCL	-0.09	-3.33	0		0.46	0.36	0
Ramelteon	0.17	-2.3	0		0.35	0.19	0
Marbofloxacin	-0.07	-3.86	0		0.17	0.59	0
Flurbiprofen (Ansaid)	-1.27	-3.02	0		0.11	0.64	0
Gemcitabine (Gemzar)	0	-4.01	0		0.07	0.75	0
Cefdinir (Omnicef)	-2	-5.63	0		0.05	0.58	0
Pitavastatin calcium (Livalo)	-0.94	-3.72	0		0.11	0.66	0
Irinotecan	0.67	-3.66	0		0.84	0.45	1
Glipizide (Glucotrol)	-1.28	-4.19	0		0.77	0.34	1
Crizotinib	0.52	-3.8	0		0.52	0.31	0
Methylprednisolone	-0.32	-2.66	1	OATP2	0.92	0.87	1
Drospirenone	-0.29	-2.51	0		0.47	0.18	0
Lamivudine (Epivir)	-0.04	-3.51	0		0.06	0.71	0
Sulfapyridine (Dagenan)	-0.47	-2.87	0		0.08	0.78	0
Vismodegib	-0.52	-2.52	0		0.15	0.32	0
Bendamustine HCL	-0.94	-2.94	0		0.05	0.65	0
Ruxolitinib	0	-2.4	0		0.2	0.13	0
Leucovorin Calcium	-2	-7.62	1	Nutrient trasnporters	0.05	0.57	0
Alfuzosin hydrochloride (Uroxatral)	-0.87	-4.01	0		0.84	0.31	1
Sumatriptan succinate	0.13	-3.29	1	Organic cation	0.16	0.58	0
Adefovir Dipivoxil (Preveon, Hepsera)	0.67	-3.26	0		0.91	0.74	1
Pyridostigmine Bromide (Mestinon)	-0.02	-4.77	1	OCTN2	0.12	0.61	0
Gemfibrozil (Lopid)	0.18	-3.17	0		0.11	0.65	0
Sulfameter (Bayrena)	-0.59	-3.81	0		0.08	0.65	0
Nitazoxanide (Alinia, Annita)	-1.09	-3.13	0		0.1	0.37	0
Ketorolac (Toradol)	-1.32	-3.16	0		0.08	0.41	0
Floxuridine	0	-4.01	1	Nutrient trasnporters	0.07	0.86	0
Dexamethasone	0.01	-2.66	0		0.94	0.88	1
Flumazenil	-0.1	-2.25	0		0.15	0.54	0
Nelarabine (Arranon)	-0.07	-4.35	1	Nutrient trasnporters	0.06	0.68	0
Etoposide (VP-16)	-1.06	-3.69	0		0.79	0.64	1
Nepafenac	-0.45	-2.74	0		0.27	0.24	0
Tianeptine sodium	-0.45	-3.2	0		0.4	0.11	0
Aminoglutethimide (Cytadren)	0.13	-2.56	0		0.3	0.29	0
Norfloxacin (Norxacin)	-0.08	-3.9	0		0.36	0.57	0
Apixaban	-0.82	-3.01	0		0.64	0.05	0
Thiabendazole	-0.15	-2.16	0		0.07	0.42	0
Amprenavir (Agenerase)	0.24	-3.33	0		0.87	0.42	1
Adenosine (Adenocard)	0.01	-4.27	1	Nutrient trasnporters	0.03	0.69	0

Ftorafur	-0.05	-2.91	0		0.09	0.69	0
Capecitabine (Xeloda)	0.05	-4.72	0		0.14	0.53	0
Cefoselis sulfate	-2	-6.2	0		0.07	0.49	0
Evista (Raloxifene Hydrochloride)	0.25	-3.82	0		0.78	0.44	1
Ondansetron hydrochloride (Zofran)	-0.08	-2.3	0		0.69	0.6	0
Prazosin HCl	-1.13	-3.04	0		0.67	0.45	0
Tizanidine HCl	-0.06	-2.66	1	Organic cation	0.07	0.42	0
Aminophylline (Truphylline)	-0.06	-2.64	0		0.06	0.51	0
Azathioprine (Azasan, Imuran)	-0.12	-3.04	0		0.05	0.39	0
Darunavir	0.62	-3.43	0		0.95	0.44	1
Guaifenesin (Guaiphenesin)	-0.44	-2.43	0		0.07	0.48	0
Daptomycin	-2	-15.01	0		0.8	0.54	0
Agomelatine	0.07	-2.37	0		0.16	0.41	0
Indomethacin (Indocid, Indocin)	-0.96	-3.36	0		0.16	0.62	0
Chlorothiazide	-1.06	-4.37	0		0.11	0.52	0
Zolmitriptan (Zomig)	0.53	-3.19	1	Organic cation	0.66	0.46	0
Piroxicam (Feldene)	-1.58	-3.73	0		0.17	0.13	0
Hydrochlorothiazide	-0.34	-3.41	0		0.29	0.42	0
Deferasirox	-0.2	-4	0		0.03	0.23	0
Rifampin	-2	-5.5	0		0.89	0.52	1

Table S3: ADME predictors of intestinal absorption and bioavailability for analysed drugs.

Name – The drug name – supplied with the library.
LogD (pH = 7.40) – The lipophilicity metric calculated from consensus $logP_{ow}$ value, considering ionization state at pH 7.4 - calculated with Percepta.
Pe (Jejunum) – Calculated Jejunal epithelial cell permeability coefficient at pH 6.5. In units of $*10^{-4}$ cm/s - calculated with Percepta.
Pe (Caco-2) with LogD – Calculated Caco2 cell permeability coefficient under stirred conditions. Determined from LogD. In units of $*10^{-6}$ cm/s (fixed conditions: pH = 7.40, rpm = 500.00) - calculated with Percepta.
Bioavailability – Calculated percent fraction that reaches systemic circulation according to dosage. Expressed as percentage (fixed conditions: Dose, mg = 10.00) - calculated with Percepta.
Maximum passive absorption (%) – Calculated estimate of maximal intestinal passive absorption. Considered from relative contributions of transcellular route to absorption (%) transcellular and paracellular route to absorption (%). Calculated from chemical structure, lipophilicity and ionization constants with Percepta. Expressed as a percentage.

Name	LogD (pH = 7.40)	Pe (Jejunum), 10^-4 cm/s	Pe (Caco-2) with LogD, 10^-6 cm/s (pH = 7.40, rpm = 500.00)	Bioavailability, % (Dose, mg = 10.00)	Maximum passive absorption (%)	Contribution of transcellular route to absorption (%)	Contribution of paracellular route to absorption (%)
Topiramate	2.14	7.77	82.92	99.44	100	99.99	0.01
Ifosfamide	0.68	7.14	55.79	99.6	100	99.91	0.09
Rufinamide (Banzel)	0.42	4.77	20.89	98.98	99.99	99.54	0.46
Fluoxetine HCl	1.71	7.9	111.96	99.33	100	99.98	0.02
Ipratropium bromide	-1.62	0.03	4.5	4.7	9.11	0.01	99.99
Ubenimex (Bestatin)	-1.39	0.14	0.34	22.19	39.01	93.57	6.43
Fluconazole	0.7	6.93	54.71	99.24	100	99.94	0.06
Anastrozole	2.67	7.91	199.06	99.22	100	100	0
Saxagliptin	-0.28	0.28	3.61	38.89	61.67	85.17	14.83
Pemetrexed	-4.47	0	0.14	0.02	0.04	14.89	85.11
Doxorubicin (Adriamycin)	-0.56	0.01	0.18	2.01	3.99	78.15	21.85
Leflunomide	2.2	8.74	145.68	99.18	100	99.99	0.01
Repaglinide	2.01	6.56	67.95	99.41	100	100	0
Ofloxacin (Floxin)	-1.5	0.89	5.29	74.97	93.3	98.43	1.57
Chloramphenicol (Chloromycetin)	0.86	4.08	12.83	98.87	99.97	99.74	0.26
Dyphylline (Dilor)	-0.94	1	4.82	80.67	94.96	97.15	2.85
Olmesartan medoxomil (Benicar)	2.12	5.65	71.38	99.41	99.99	100	0
Nevirapine (Viramune)	2.1	8.32	137.83	99.08	100	99.99	0.01
Cefditoren pivoxil	2.45	6.19	42.33	91.96	100	100	0
Etodolac (Lodine)	0.41	5.16	18.1	99.32	99.99	99.98	0.02
Dorzolamide HCL	-0.35	0.44	3.59	53.37	76.64	89.29	10.71
Ramelteon	2.8	8.26	161.4	98.59	100	100	0
Marbofloxacin	-1.94	0.44	3.32	53.22	76.92	93.56	6.44
Flurbiprofen (Ansaid)	0.68	7.5	54.94	99.23	100	99.99	0.01
Gemcitabine (Gemzar)	-1.36	0.13	1.04	20.81	36.8	68.27	31.73
Cefdinir (Omnicef)	-4.51	0	0.16	0.35	0.71	29.71	70.3
Pitavastatin calcium (Livalo)	0.45	3.38	7.15	97.99	99.92	99.99	0.01
Irinotecan	1.86	6.24	107.91	99.17	100	100	0
Glipizide (Glucotrol)	0.01	1.45	4.34	89.13	98.15	99.97	0.03
Crizotinib	2.05	6.57	35.3	99.28	100	99.98	0.02
Methylprednisolone	1.97	6.45	33.78	99.59	100	99.99	0.01
Drospirenone	3.47	7.55	194.64	70.3	100	100	0
Lamivudine (Epivir)	-0.95	0.45	2.47	54.57	77.78	90.31	9.69
Sulfapyridine (Dagenan)	0.43	2.83	8.7	96.79	99.84	99.23	0.77
Vismodegib	3.13	7.5	156.71	14.79	100	100	0
Bendamustine HCL	0.25	6.6	33.42	99.63	100	99.98	0.02
Ruxolitinib	2.31	7.9	144.6	99.43	100	100	0
Leucovorin Calcium	-7.14	0	0.13	0.01	0.02	0.15	99.85
Alfuzosin hydrochloride (Uroxatral)	-1.02	0.28	1.5	38.84	61.48	93.6	6.4
Sumatriptan succinate	-0.73	1.01	5.42	80.42	94.98	95.6	4.4
Adefovir Dipivoxil (Preveon, Hepsera)	2.2	6.65	76.8	99.73	100	100	0
Pyridostigmine Bromide (Mestinon)	-3.34	0.16	3.3	25.22	44.26	0	100
Gemfibrozil (Lopid)	1.58	8	104.81	99.46	100	100	0
Sulfameter (Bayrena)	-0.49	1.6	3.1	90.68	98.64	99.19	0.81
Nitazoxanide (Alinia, Annita)	1.01	7.97	71.07	99.76	100	99.99	0.01
Ketorolac (Toradol)	-0.93	3.12	10.82	98.1	99.89	99.91	0.09
Floxuridine	-1.56	0.25	1.45	35.84	58.41	83.97	16.03
Dexamethasone	1.92	6.37	32.38	99.35	100	99.99	0.01
Flumazenil	1	7.9	122.05	99.79	100	99.99	0.01
Nelarabine (Arranon)	-0.98	0.09	0.62	14.94	27.58	72.45	27.55
Etoposide (VP-16)	0.66	3.11	8.36	97.95	99.89	99.99	0.01
Nepafenac	1.38	3.94	9.53	98.76	99.96	99.67	0.33
Tianeptine sodium	0.67	5.96	21.33	98.63	100	99.99	0.01
Aminoglutethimide (Cytadren)	1.27	5.55	20.41	99.36	99.99	99.8	0.2
Norfloxacin (Norxacin)	-3.08	0.1	0.59	16.62	30.7	72.43	27.57
Apixaban	0.87	5.27	25.72	99.3	99.99	99.99	0.01
Thiabendazole	2.39	9.36	157.6	99.4	100	99.99	0.01
Amprenavir (Agenerase)	3.22	6.49	22.45	96.64	100	100	0
Adenosine (Adenocard)	-1.05	0.09	0.73	15.08	27.83	63.95	36.05

Ftorafur	-0.59	3.81	17.99	99.11	99.96	98.96	1.04
Capecitabine (Xeloda)	-1.34	0.08	1.18	13.63	25.32	97.64	2.36
Cefoselis sulfate	-3.98	0	0.14	0.56	1.11	31.13	68.87
Evista (Raloxifene Hydrochloride)	4.27	6.93	55.81	98.81	100	100	0
Ondansetron hydrochloride (Zofran)	2.53	7.94	197.21	99.33	100	99.99	0.01
Prazosin HCl	0.96	3.79	29.87	98.91	99.96	99.71	0.29
Tizanidine HCl	0.95	3.15	35.07	98.21	99.9	97.57	2.43
Aminophylline (Truphylline)	0.1	6.05	36.39	99.54	100	99.51	0.49
Azathioprine (Azasan, Imuran)	-0.04	5.2	28.35	99.25	99.99	99.74	0.26
Darunavir	3.26	6.39	21.81	97.11	100	100	0
Guaifenesin (Guaiphenesin)	0.84	6.27	31.93	99.62	100	99.76	0.24
Daptomycin	-9.71	0	0.09	0	0	100	0
Agomelatine	3.02	8.44	163.87	98.76	100	100	0
Indomethacin (Indocid, Indocin)	0.74	6.95	53.3	99.4	100	100	0
Chlorothiazide	-1.41	0.4	1.37	50.79	74.35	97.1	2.9
Zolmitriptan (Zomig)	0.29	3.45	16.1	98.32	99.93	99.12	0.88
Piroxicam (Feldene)	-1.05	0.96	3.74	79	94.36	99.74	0.27
Hydrochlorothiazide	0	0.77	2.55	71.81	90.82	95.99	4.01
Deferasirox	1.07	4.66	14.66	98.93	99.98	99.99	0.01
Rifampin	0	0.11	0.32	17.38	31.61	100	0

Table S4: Bulk loss analysis for analyzed drugs.

Name – The drug name – supplied with the library.
Method – Whether the drug was analyzed by mass spectrometry (MSD) or via HPLC (UV).
MSD retention time - aqueous – The retention time of the MSD peak of the drug in the initial aqueous sample.
MSD area - aqueous – The MSD peak area of the drug in the initial aqueous sample.
MSD retention time – two phase – The retention time of the MSD peak of the drug in the second measurement of the sample after incubation as a hexadecane - water partition.
MSD area – two phase – The MSD peak area of the drug in the second measurement of the sample after incubation as an hexadecane - water partition.
UV280 retention time - aqueous – The retention time of the UV peak of the drug in the initial aqueous sample.
UV280 area - aqueous – The UV peak area of the drug in the initial aqueous sample.
UV280 retention time – two phase – The retention time of the UV peak of the drug in the second measurement of the sample after incubation as an hexadecane - water partition.
UV280 area – two phase – The UV peak area of the drug in the second measurement of the sample after incubation as an hexadecane - water partition.
Signal loss MSD – The signal loss between the two runs in MSD or UV signal according to Method. Expressed as a percentage. Set to 0 in instances where signal was higher in the second run. In Figure 2 b, the area values were plotted without this correction. As stated throughout, drug concentrations were assessed in the MSD only if they did not absorb in the UV. The MSD was used to assign all peaks where possible.

Name	Method	MSD retention time - aqueous	MSD area - aqueous	MSD retention time – two phase	MSD area – two phase	UV280 retention time - aqueous	UV280 area - aqueous	UV280 retention time – two phase	UV280 area – two phase	Signal loss, %
Topiramate	MSD	5.39	1957500.00	5.37	1560000.00					20.31
Ifosfamide	MSD	4.84	3645060.00	4.82	2360000.00					35.25
Rufinamide (Banzel)	MSD	4.51	1872610.00	4.51	2230000.00					0.00
Fluoxetine HCl	MSD	5.94	10896062.00	5.95	2175640.00					80.03
Ipratropium bromide	MSD	4.32	15086300.00	5.08	16073100.00					0.00
Ubenimex (Bestatin)	MSD	4.58	11992700.00	4.70	8938760.00					25.46
Fluconazole	MSD	4.96	6308760.00	4.49	8980940.00					0.00
Anastrozole	MSD	5.93	9359290.00	5.91	5840000.00					37.60
Saxagliptin	MSD	4.43	6588440.00	4.17	6612150.00					0.00
Pemetrexed	UV					4.16	20.71	4.14	19.45	6.08
Doxorubicin (Adriamycin)	UV	5.08	2677683.00	5.06	1955371.00	5.04	81.88	5.02	55.39	32.35
Leflunomide	UV					7.58	69.82	7.60	29.00	58.46
Repaglinide	UV	6.90	35946828.00	6.89	32564674.00	6.85	25.79	6.86	22.10	14.30
Ofloxacin (Floxin)	UV	3.79	5650222.00	3.79	4786554.00	3.75	132.78	3.75	115.59	12.95
Chloramphenicol (Chloromycetin)	UV					5.12	160.87	5.12	143.60	10.73
Dyphylline (Dilor)	UV					3.28	142.15	3.28	131.36	7.59
Olmesartan medoxomil (Benicar)	UV					6.17	49.30	5.00	26.20	46.86
Nevirapine (Viramune)	UV	5.01	11988185.00	5.00	9493000.00	4.97	116.37	4.97	103.76	10.84
Cefditoren pivoxil	UV	4.36	2075430.00	4.36	3809536.00	4.33	35.15	4.33	64.29	0.00
Etodolac (Lodine)	UV	8.78	4518137.00	8.82	1893962.00	8.74	172.68	8.76	207.37	0.00
Dorzolamide HCL	UV					2.90	15.80	3.97	24.60	0.00
Ramelteon	UV					6.82	27.59	6.18	23.80	13.75
Marbofloxacin	UV					3.84	108.52	4.66	156.94	0.00
Flurbiprofen (Ansaid)	UV					7.84	60.05	8.56	80.71	0.00
Gemcitabine (Gemzar)	UV					1.57	230.90	2.28	249.60	0.00
Cefdinir (Omnicef)	UV					3.78	352.28	4.62	472.01	0.00
Pitavastatin calcium (Livalo)	UV					6.06	145.95	6.69	193.73	0.00
Irinotecan	UV	5.15	16935700.00	6.17	6214789.00	5.09	55.50	6.10	92.80	0.00
Glipizide (Glucotrol)	UV	7.74	3630980.00	6.45	4453870.00	6.41	61.31	7.70	56.64	7.60
Crizotinib	UV	4.37	5539020.00	4.27	5906560.00	4.33	61.43	4.23	59.80	2.65
Methylprednisolone	UV	5.84	3130000.00	5.84	3210000.00	5.80	31.24	5.79	28.69	8.16
Drospirenone	UV					7.24	9.25	7.20	14.27	0.00
Lamivudine (Epivir)	UV	2.04	1811300.00	1.97	2752091.00	1.99	135.07	1.92	223.80	0.00
Sulfapyridine (Dagenan)	UV	3.59	2315750.00	3.58	1629450.00	3.54	64.51	3.53	53.05	17.77
Vismodegib	UV					9.08	132.40	6.55	128.32	3.08
Bendamustine HCL	UV	3.36	3527020.00	3.24	4634320.00	3.32	24.89	3.19	26.80	0.00
Ruxolitinib	UV	5.46	9103790.00	5.27	13846300.00	5.41	111.50	5.23	120.75	0.00
Leucovorin Calcium	UV					3.45	169.70	3.24	143.99	15.15
Alfuzosin hydrochloride (Uroxatral)	UV	4.42	14357100.00	4.41	16771000.00	4.37	83.39	4.36	81.52	2.25
Sumatriptan succinate	UV	3.32	6916410.00	3.35	7912840.00	3.27	79.63	3.30	82.73	0.00
Adefovir Dipivoxil (Preveon, Hepsera)	UV	3.79	7193870.00	3.86	7259070.00	3.74	48.33	3.81	51.39	0.00
Pyridostigmine Bromide (Mestinon)	UV	1.58	5190520.00	1.71	5196228.00	1.55	55.52	1.64	51.56	7.14
Gemfibrozil (Lopid)	UV					8.64	35.27	8.67	23.17	34.30
Sulfameter (Bayrena)	UV	4.27	4183860.00	4.27	3626070.00	4.22	232.19	4.22	189.63	18.33
Nitazoxanide (Alinia, Annita)	UV					7.00	33.71	7.00	29.91	11.28
Ketorolac (Toradol)	UV	6.25	3287800.00	6.26	3524790.00	6.21	85.62	6.22	87.05	0.00
Floxuridine	UV					1.94	103.37	1.99	108.37	0.00
Dexamethasone	UV	5.93	1320000.00	5.89	1094000.00	5.88	25.81	5.85	25.08	2.83

Flumazenil	UV	5.11	8550000.00	5.09	9361000.00	5.06	29.86	5.05	32.28	0.00
Nelarabine (Arranon)	UV	2.82	1359000.00	2.81	3970000.00	2.77	124.93	2.76	137.95	0.00
Etoposide (VP-16)	UV	5.33	3024473.00	5.32	2351746.00	5.29	53.34	5.28	44.36	16.84
Nepafenac	UV	5.71	6320000.00	5.70	6290000.00	5.67	70.77	5.66	76.22	0.00
Tianeptine sodium	UV	7.23	8560000.00	7.22	8822700.00	7.18	46.76	7.17	46.38	0.81
Aminoglutethimide (Cytadren)	UV	3.64	4814190.00	3.64	5155000.00	3.59	2.12	3.59	2.74	0.00
Norfloxacin (Norxacin)	UV	3.57	5921000.00	3.56	485000.00	3.52	385.36	3.50	413.00	0.00
Apixaban	UV	5.66	1634000.00	5.65	5549000.00	5.62	147.58	5.61	141.63	4.03
Thiabendazole	UV	3.47	3928000.00	3.46	2954000.00	3.42	99.13	3.42	73.49	25.87
Amprenavir (Agenerase)	UV	6.81	14016000.00	6.80	13662300.00	6.76	134.46	6.75	136.59	0.00
Adenosine (Adenocard)	UV	2.17	1122000.00	2.16	1008690.00	2.18	17.15	2.11	17.37	0.00
Ftorafur	UV					3.35	121.95	3.33	114.74	5.91
Capecitabine (Xeloda)	UV	5.45	7874480.00	5.44	6725150.00	5.40	51.97	5.40	53.79	0.00
Cefoselis sulfate	UV	2.76	5133260.00	2.75	4524480.00	2.71	186.53	2.70	179.58	3.72
Evista (Raloxifene Hydrochloride)	UV	5.08	1981620.00	5.08	1866930.00	5.03	44.67	5.02	42.38	5.13
Ondansetron hydrochloride (Zofran)	UV	4.39	5616300.00	4.38	5376420.00	4.35	36.78	4.34	39.08	0.00
Prazosin HCl	UV	4.22	7043860.00	4.22	7330000.00	4.17	137.06	4.17	142.32	0.00
Tizanidine HCl	UV	2.98	5401230.00	2.97	5400000.00	2.92	21.67	2.93	21.65	0.07
Aminophylline (Truphylline)	UV					3.01	207.73	3.02	237.76	0.00
Azathioprine (Azasan, Imuran)	UV	3.50	1838950.00	3.49	1490000.00	3.45	224.84	3.45	231.97	0.00
Darunavir	UV	6.79	5.93	6.78	6780000.00	6.75	65.74	6.73	68.46	0.00
Guaifenesin (Guaiphenesin)	UV	4.27	1242920.00	4.27	1140000.00	4.22	22.65	4.22	24.28	0.00
Daptomycin	UV					6.13	20.72	6.13	19.96	3.70
Agomelatine	UV	6.29	4060910.00	6.27	2660000.00	6.24	44.26	6.22	13.67	69.11
Indomethacin (Indocid, Indocin)	UV	7.70	2240000.00	7.69	1870000.00	7.66	127.90	7.65	118.83	7.09
Chlorothiazide	UV					2.94	105.12	2.93	109.35	0.00
Zolmitriptan (Zomig)	UV					3.18	60.53	3.19	64.39	0.00
Piroxicam (Feldene)	UV	6.36	14635221.00	6.36	13547470.00	6.32	114.71	6.32	107.43	6.34
Hydrochlorothiazide	UV					3.15	153.43	3.13	173.16	0.00
Deferasirox	UV	7.56	9440000.00	7.56	12300000.00	7.51	209.26	7.52	201.04	3.93
Rifampin	UV	7.10	12800000.00	7.09	11300000.00	7.06	119.49	7.04	96.22	19.47

Table S5: Drug mixture analysis for measured drugs.

Name – The drug name – supplied with the library.
Method – Whether the drug was analyzed by mass spectrometry (MSD) or via HPLC (UV).
DIB mixture number – An indicator of the mixture the drug was analysed in.
MSD area - aqueous – The MSD peak area of the drug in the initial aqueous sample.
MSD Area Dt=0 – The MSD peak area of the drug in the donor droplets prior to DIB formation (the initial concentration).
MSD Area Dt=16 – The MSD peak area of the drug in the donor droplets after 16 h incubation in DIBs.
MSD Area At=16 – The MSD peak area of the drug in the acceptor droplets after 16 h incubation in DIBs.
UV280 Area Dt=0 – The UV peak area of the drug in the donor droplets prior to DIB formation (the initial concentration).
UV280 Area Dt=16 – The UV peak area of the drug in the donor droplets after 16 h incubation in DIBs.
UV280 Area At=16 – The UV peak area of the drug in the acceptor droplets after 16 h incubation in DIBs.
MSD confirmed mass – Confirmation of whether the corresponding drug mass signature was detected in the MSD at the retention time and thereby used to confirm mass for each experimental chromatogram. Note that not all MSD peaks could be integrated reliably, however their mass peak distribution could still be assessed. Some drugs did not demonstrate any mass signal in the MSD.
Signal lost – The overall signal lost in the system according to Method. Used to calculate Loss classifier.
Permeability classifier – Determined accoring to peak area distribution and Method. See equation 1.
Loss classifier – Determined according to Signal lost. See equation 2.

Name	Method	DIB mixture number	MSD Area Dt=0	MSD Area Dt=16	MSD Area At=16	UV280 Area Dt=0	UV280 Area Dt=16	UV280 Area At=16	MSD confirmed mass	Signal lost, %	Permeability classifier	Loss classifier
Topiramate	MSD	3	990134.00	507051.00	462337.00				Yes	2.10	1.00	0.00
Ifosfamide	MSD	11	782830.00	603720.00	420233.00				Yes	0.00	0.50	0.00
Rufinamide (Banzel)	MSD	7	1207990.00	325001.00	292597.00				Yes	48.87	1.00	0.50
Fluoxetine HCl	MSD	8	3729280.00	721531.00	443568.00				Yes	68.76	0.50	1.00
Ipratropium bromide	MSD	2	6833694.00	4767740.00	0.00				Yes	30.23	0.00	0.50
Ubenimex (Bestatin)	MSD	1	1355760.00	1451930.00	0.00				Yes	0.00	0.00	0.00
Fluconazole	MSD	9	1339790.00	691737.00	680820.00				Yes	0.00	1.00	0.00
Anastrozole	MSD	7	2028050.00	609776.00	634035.00				Yes	38.67	1.00	0.50
Saxagliptin	MSD	4	906656.00	363658.00	343060.00				Yes	22.05	1.00	0.50
Pemetrexed	UV	1	429902.00	311060.00	0.00	16.50	15.90	0.00	Yes	3.64	0.00	0.00
Doxorubicin (Adriamycin)	UV	2				96.39	63.16	21.17	Yes	12.51	0.50	0.00
Leflunomide	UV	12				21.73	2.74	2.42	Yes	76.26	1.00	1.00
Repaglinide	UV	10	16708800.00	9011766.00	10113215.00	19.13	9.08	10.73	Yes	0.00	1.50	0.00
Ofloxacin (Floxin)	UV	3				59.44	24.52	25.78	Yes	15.37	1.00	0.00
Chloramphenicol (Chloromycetin)	UV	8				6.70	2.56	2.79	No	20.11	1.00	0.50
Dyphylline (Dilor)	UV	2	514264.00	275407.00	212320.00	64.46	35.25	34.07	Yes	0.00	1.00	0.00
Olmesartan medoxomil (Benicar)	UV	10	4112570.00	1954040.00	1274060.00	14.28	6.09	4.95	Yes	22.68	0.50	0.50
Nevirapine (Viramune)	UV	9	2565510.00	610479.00	444057.00	37.69	7.96	6.32	Yes	62.11	0.50	1.00
Cefditoren pivoxil	UV	12	665461.00	673589.00	0.00	29.60	29.48	0.00	Yes	0.39	0.00	0.00
Etodolac (Lodine)	UV	12				31.12	13.72	12.68	Yes	15.15	1.00	0.00
Dorzolamide HCL	UV	7	545892.00	255639.00	335350.00	3.73	1.73	1.78	Yes	6.05	1.00	0.00
Ramelteon	UV	11				5.24	1.08	1.05	Yes	59.44	1.00	1.00
Marbofloxacin	UV	8				16.18	6.37	7.03	Yes	17.15	1.00	0.00
Flurbiprofen (Ansaid)	UV	5				37.94	18.69	18.35	No	2.36	1.00	0.00
Gemcitabine (Gemzar)	UV	9				24.93	24.64	0.00	Yes	1.13	0.00	0.00
Cefdinir (Omnicef)	UV	6	705723.00	769733.00	0.00	118.39	116.33	0.00		1.74	0.00	0.00
Pitavastatin calcium (Livalo)	UV	8	9273400.00	4356520.00	3614370.00	32.40	13.84	12.63	Yes	18.31	1.00	0.00
Irinotecan	UV	1	1290920.00	690465.00	420970.00	5.52	3.18	2.40	Yes	0.00	0.50	0.00
Glipizide (Glucotrol)	UV	10	3928605.00	4360150.00	477285.00	15.91	15.74	2.23	Yes	0.00	0.50	0.00
Crizotinib	UV	6	3429990.00	1613310.00	728013.00	40.76	19.96	7.90	Yes	31.65	0.50	0.50
Methylprednisolone	UV	7				9.90	3.85	1.23	Yes	48.67	0.50	0.50
Drospirenone	UV	7				20.04	1.75	1.63	Yes	83.14	1.00	1.00
Lamivudine (Epivir)	UV	10	887122.00	812262.00	0.00	22.69	18.97	0.00	Yes	16.39	0.00	0.00
Sulfapyridine (Dagenan)	UV	1	523006.00	278343.00	321051.00	33.97	15.98	16.82	Yes	3.44	1.00	0.00
Vismodegib	UV	5	3036670.00	1501570.00	1443122.00	110.33	50.56	52.60	Yes	6.50	1.00	0.00
Bendamustine HCL	UV	1	555372.00	569841.00	0.00	5.22	5.03	0.00	Yes	3.61	0.00	0.00
Ruxolitinib	UV	12	3301100.00	749016.00	769208.00	52.85	16.49	15.01	Yes	40.40	1.00	0.50

Leucovorin Calcium	UV	9				47.15	47.46	0.00		0.00	0.00	0.00
Alfuzosin hydrochloride (Uroxatral)	UV	9	4014440.00	2468560.00	2148070.00	29.93	16.49	11.58	Yes	6.21	0.50	0.00
Sumatriptan succinate	UV	6	2172724.00	1650490.00	546515.00	34.26	26.16	6.51	Yes	4.64	0.50	0.00
Adefovir Dipivoxil (Preveon, Hepsera)	UV	7				7.77	4.21	2.80	Yes	9.78	0.50	0.00
Pyridostigmine Bromide (Mestinon)	UV	12	612732.00	640196.00	0.00	12.10	12.85	0.00	Yes	0.00	0.00	0.00
Gemfibrozil (Lopid)	UV	8				6.12	1.25	1.33	No	58.03	1.00	1.00
Sulfameter (Bayrena)	UV	5	2654390.00	1399350.00	1134430.00	231.84	114.38	111.74	Yes	2.46	1.00	0.00
Nitazoxanide (Alinia, Annita)	UV	8				8.80	3.17	3.24	No	27.23	1.00	0.50
Ketorolac (Toradol)	UV	6				52.30	14.93	18.67	Yes	35.76	1.00	0.50
Floxuridine	UV	8				36.66	32.11	0.00	No	12.42	0.00	0.00
Dexamethasone	UV	3				8.81	3.67	3.47	Yes	18.89	1.00	0.00
Flumazenil	UV	5	6294690.00	2361459.00	3264770.00	32.69	12.27	12.11	Yes	25.43	1.00	0.50
Nelarabine (Arranon)	UV	2	1126386.00	1251191.00		76.11	71.92	0.00	Yes	5.50	0.00	0.00
Etoposide (VP-16)	UV	1				10.10	4.10	5.50	Yes	4.95	1.00	0.00
Nepafenac	UV	2				16.01	4.21	5.31	Yes	40.48	1.00	0.50
Tianeptine sodium	UV	11	2931410.00	1429480.00	1571076.00	21.15	8.18	8.99	Yes	18.83	1.00	0.00
Aminoglutethimide (Cytadren)	UV	6				5.89	5.07	2.92	Yes	0.00	0.50	0.00
Norfloxacin (Norxacin)	UV	8				81.15	37.04	29.22	Yes	18.35	0.50	0.00
Apixaban	UV	5	1506940.00	910258.00	935023.00	35.21	17.18	16.94	Yes	3.11	1.00	0.00
Thiabendazole	UV	10	1452660.00			29.79	1.91	1.92	Yes	87.17	1.00	1.00
Amprenavir (Agenerase)	UV	3	5181120.00	2206050.00	2100980.00	77.65	27.11	27.47	Yes	29.71	1.00	0.50
Adenosine (Adenocard)	UV	11	942169.00	664385.00	0.00	32.94	23.56	0.00	Yes	28.46	0.00	0.50
Ftorafur	UV	12				33.89	18.52	16.51	No	0.00	1.00	0.00
Capecitabine (Xeloda)	UV	9	2888330.00	1294840.00	1322240.00	22.32	11.67	10.58	Yes	0.30	1.00	0.00
Cefoselis sulfate	UV	3	1588770.00	1809561.00	0.00	116.22	104.42	0.00	Yes	10.16	0.00	0.00
Evista (Raloxifene Hydrochloride)	UV	11				152.82	70.63	37.59	Yes	29.19	0.50	0.50
Ondansetron hydrochloride (Zofran)	UV	3	1792880.00	465930.00	250676.00	21.59	4.45	3.93	Yes	61.16	1.00	1.00
Prazosin HCl	UV	10	3036170.00	815402.00	767075.00	81.41	20.57	19.55	Yes	50.71	1.00	1.00
Tizanidine HCl	UV	5	6701000.00	3026081.00	3397540.00	37.67	14.94	15.10	Yes	20.25	1.00	0.50
Aminophylline (Truphylline)	UV	11				140.09	59.84	61.81	Yes	13.16	1.00	0.00
Azathioprine (Azasan, Imuran)	UV	5	1898830.00	1492870.00	1134430.00	362.80	164.71	172.54	Yes	7.04	1.00	0.00
Darunavir	UV	6	4985870.00	1681460.00	1978430.00	139.69	27.79	29.02	Yes	59.33	1.00	1.00
Guaifenesin (Guaiphenesin)	UV	11				15.11	6.74	6.46	Yes	12.64	1.00	0.00
Daptomycin	UV	7				17.40	20.80	0.00	Yes	0.00	0.00	0.00
Agomelatine	UV	9	1821310.00	150261.00	173838.00	22.63	2.82	2.73	Yes	75.51	1.00	1.00
Indomethacin (Indocid, Indocin)	UV	6				75.53	20.25	27.31	Yes	37.03	1.00	0.50
Chlorothiazide	UV	10				104.79	101.77	0.00	Yes	2.88	0.00	0.00
Zolmitriptan (Zomig)	UV	12				35.24	24.37	10.64	Yes	0.66	0.50	0.00
Piroxicam (Feldene)	UV	4	1362810.00	565572.00	546619.00	54.50	13.90	13.40	Yes	49.91	1.00	0.50
Hydrochlorothiazide	UV	4				36.20	34.51	0.00	No	4.68	0.00	0.00
Deferasirox	UV	4	520192.00	76796.00	71724.00	47.10	3.39	3.22	Yes	85.95	1.00	1.00
Rifampin	UV	4	1284890.00	235111.00	155065.00	46.20	7.48	5.99	Yes	70.86	0.50	1.00

Table S6: Retention times for measured drugs.

Name – The drug name – supplied with the library.
Method – Whether the drug was analyzed by mass spectrometry (MSD) or via HPLC (UV).
Mean retention time – Determined for chosen method – used in Figure 2 f.
Retention count – Number of individual repeats of the retention time of drugs. Note, that 4 or 5. 4 in the instance of impermeability since no addtiional retention time was recorded for the acceptor droplet.
Retention standard deviation – Determined from mean and retention count.

Name	Method	DIB mixture number	Mean retention (min)	Retention count	Retention standard deviation (min)
Topiramate	MSD	3	5.44	5	0.05
Ifosfamide	MSD	11	5.29	5	0.44
Rufinamide (Banzel)	MSD	7	4.88	5	0.34
Fluoxetine HCl	MSD	8	6.20	5	0.24
Ipratropium bromide	MSD	2	4.35	4	0.51
Ubenimex (Bestatin)	MSD	1	4.52	4	0.14
Fluconazole	MSD	9	4.84	5	0.20
Anastrozole	MSD	7	6.21	5	0.27
Saxagliptin	MSD	4	3.97	5	0.21
Pemetrexed	UV	1	3.98	4	0.19
Doxorubicin (Adriamycin)	UV	2	4.89	5	0.12
Leflunomide	UV	12	7.70	5	0.11
Repaglinide	UV	10	6.83	5	0.03
Ofloxacin (Floxin)	UV	3	3.68	5	0.07
Chloramphenicol (Chloromycetin)	UV	8	5.45	5	0.31
Dyphylline (Dilor)	UV	2	3.20	5	0.07
Olmesartan medoxomil (Benicar)	UV	10	6.01	5	0.57
Nevirapine (Viramune)	UV	9	5.24	5	0.25
Cefditoren pivoxil	UV	12	4.53	4	0.24
Etodolac (Lodine)	UV	12	8.28	5	0.43
Dorzolamide HCL	UV	7	3.26	5	0.41
Ramelteon	UV	11	6.78	5	0.35
Marbofloxacin	UV	8	4.07	5	0.34
Flurbiprofen (Ansaid)	UV	5	8.57	5	0.43
Gemcitabine (Gemzar)	UV	9	2.00	4	0.93
Cefdinir (Omnicef)	UV	6	3.82	4	0.56
Pitavastatin calcium (Livalo)	UV	8	6.19	5	0.28
Irinotecan	UV	1	5.05	5	0.61
Glipizide (Glucotrol)	UV	10	5.97	5	1.09
Crizotinib	UV	6	4.25	5	0.04
Methylprednisolone	UV	7	6.08	5	0.26
Drospirenone	UV	7	7.42	5	0.18
Lamivudine (Epivir)	UV	10	2.54	4	0.67
Sulfapyridine (Dagenan)	UV	1	3.60	5	0.06
Vismodegib	UV	5	7.84	5	0.90
Bendamustine HCL	UV	1	3.14	4	0.14
Ruxolitinib	UV	12	5.51	5	0.19
Leucovorin Calcium	UV	9	3.44	4	0.14
Alfuzosin hydrochloride (Uroxatral)	UV	9	4.56	5	0.18
Sumatriptan succinate	UV	6	3.17	5	0.11
Adefovir Dipivoxil (Preveon, Hepsera)	UV	7	3.91	5	0.13
Pyridostigmine Bromide (Mestinon)	UV	12	1.86	4	0.31
Gemfibrozil (Lopid)	UV	8	8.65	5	0.01
Sulfameter (Bayrena)	UV	5	4.80	5	0.79
Nitazoxanide (Alinia, Annita)	UV	8	7.11	5	0.11
Ketorolac (Toradol)	UV	6	6.07	5	0.13
Floxuridine	UV	8	2.32	4	0.41
Dexamethasone	UV	3	5.91	5	0.04
Flumazenil	UV	5	6.08	5	0.94
Nelarabine (Arranon)	UV	2	2.81	4	0.04
Etoposide (VP-16)	UV	1	5.34	5	0.05
Nepafenac	UV	2	5.40	5	0.04
Tianeptine sodium	UV	11	7.45	5	0.25
Aminoglutethimide (Cytadren)	UV	6	3.65	5	0.06
Norfloxacin (Norxacin)	UV	8	3.81	5	0.27
Apixaban	UV	5	6.52	5	0.84
Thiabendazole	UV	10	3.71	5	0.27
Amprenavir (Agenerase)	UV	3	6.77	5	0.02
Adenosine (Adenocard)	UV	11	2.53	4	0.46
Ftorafur	UV	12	3.77	5	0.40
Capecitabine (Xeloda)	UV	9	5.77	5	0.34
Cefoselis sulfate	UV	3	2.75	4	0.05
Evista (Raloxifene Hydrochloride)	UV	11	5.38	5	0.34
Ondansetron hydrochloride (Zofran)	UV	3	4.40	5	0.05
Prazosin HCl	UV	10	4.45	5	0.25
Tizanidine HCl	UV	5	3.93	5	0.93
Aminophylline (Truphylline)	UV	11	3.41	5	0.38
Azathioprine (Azasan, Imuran)	UV	5	4.73	5	1.17
Darunavir	UV	6	6.74	5	0.01
Guaifenesin (Guaiphenesin)	UV	11	4.66	5	0.42
Daptomycin	UV	7	6.26	4	0.16
Agomelatine	UV	9	6.58	5	0.32
Indomethacin (Indocid, Indocin)	UV	6	7.65	5	0.00
Chlorothiazide	UV	10	3.24	4	0.35
Zolmitriptan (Zomig)	UV	12	3.55	5	0.33
Piroxicam (Feldene)	UV	4	6.19	5	0.12
Hydrochlorothiazide	UV	4	3.19	5	0.07
Deferasirox	UV	4	7.55	5	0.03
Rifampin	UV	4	7.08	5	0.03

a

b

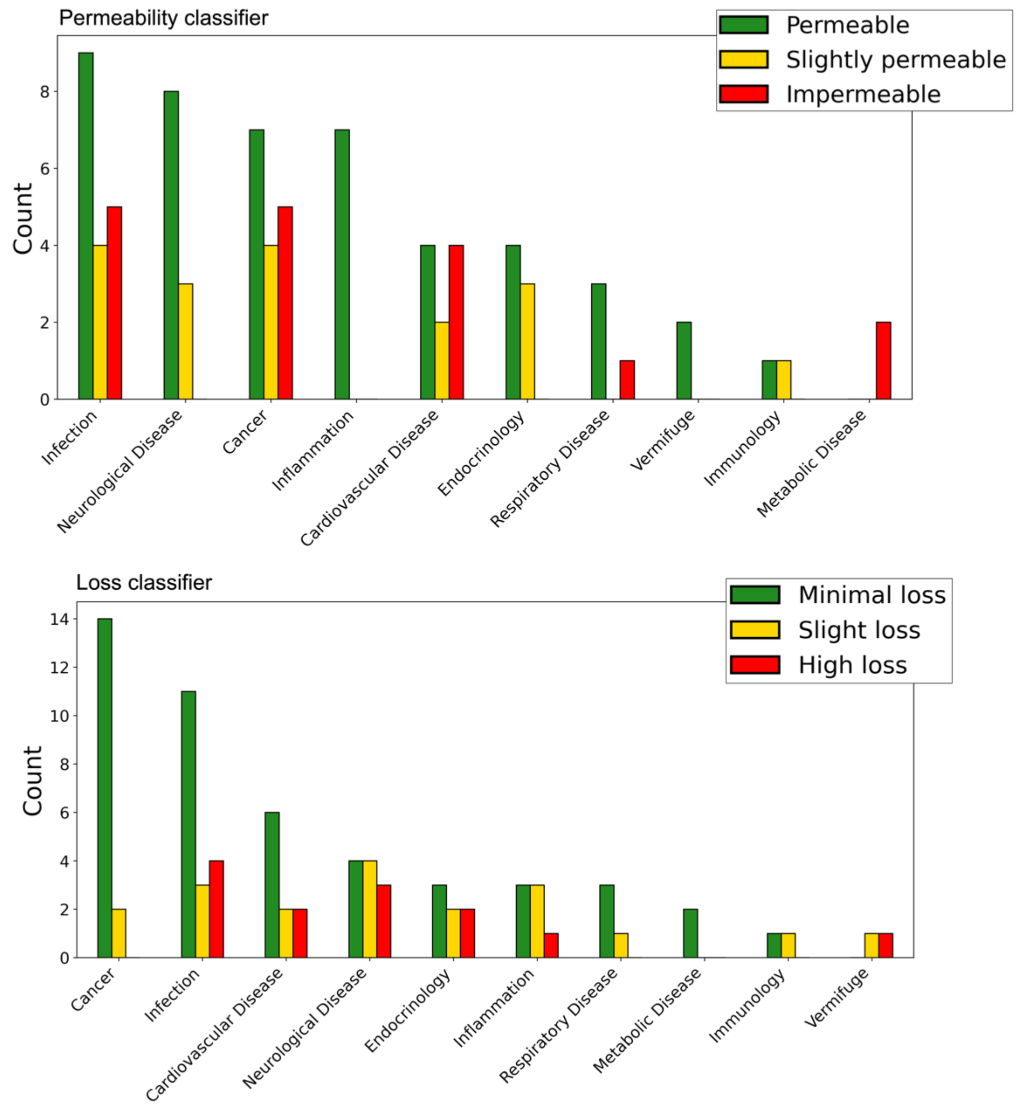

Figure S5: Indication space of classifiers. (a) The majority of indications contained permeable drugs. Of the statistically significant indications, inflammation and neurological disease exclusively contained permeable drugs. **(b)** Loss was observed across most indications. Of the statistically signifcant indications, cancer contained no high loss drugs.

Table S7: Statistical testing of physicochemical and ADME values for separation in distributions of simple diffusion permeability classifiers.

	Kruskal-Wallis (p value , H)*	Mann-Whitney (p value, U)**		
	All classifier distirbutions	Permeable vs impermeable	Slight permeable vs impermeable	Permeable vs Slight permeable
Retention time	0.000004, 25.03	0.000007, 682	0.002, 243	0.20, 499
$logD_{(pH = 7.4)}$	0.00002, 22.11	0.00001, 640	0.001,222	1,360
HBD	0.0007, 14.45	0.0008, 143	0.052, 65.50	0.54, 281
MW	0.1, 4.01	1,332	0.80, 158	0.13, 235.5
HBA	0.04, 6.52	0.033, 205	0.53, 92	1, 310
RBN	0.20, 3.25	1, 340	1, 141	0.13, 237
TPSA	0.019, 7.92	0.02, 189	0.2, 77	1, 334
logP	0.000028, 20.9	0.00005, 623	0.001, 224	1, 301
Maximum passive absorption	0.00001, 22.52	0.000008, 637	0.0042, 212	1, 383
Caco2 permeability	0.00009,23.3	0.000004, 654	0.003,216	1,386
Rat jejunem permeability	0.00009,23.2	0.000006, 651	0.0025, 217	1, 399

***Determined with python (https://docs.scipy.org/doc/scipy/reference/generated/scipy.stats.kruskal.html). Significance associated with a p value <0.05.**
****Determined with python (https://docs.scipy.org/doc/scipy/reference/generated/scipy.stats.mannwhitneyu.html). Performed with Bonferonni correction to reduce false positives. Signficance associated with a Bonfeornni corrected pvalue <0.0167.**
Colored according to significance, green = significant differences between distribtuions, red = non significant.

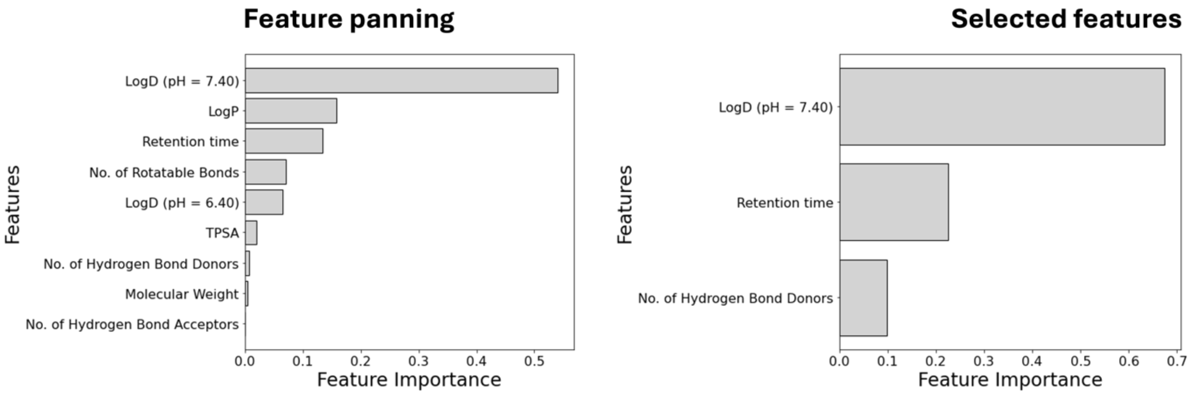


```
              precision    recall  f1-score   support

           0       0.33      0.33      0.33         3
           1       0.80      0.80      0.80        10

    accuracy                           0.69        13
   macro avg       0.57      0.57      0.57        13
weighted avg       0.69      0.69      0.69        13

Confusion Matrix:
[[1 2]
 [2 8]]
```

```
              precision    recall  f1-score   support

           0       0.50      0.67      0.57         3
           1       0.89      0.80      0.84        10

    accuracy                           0.77        13
   macro avg       0.69      0.73      0.71        13
weighted avg       0.80      0.77      0.78        13

Confusion Matrix:
[[2 1]
 [2 8]]
```

Figure S6: Extreme gradient boosting (XGBOOST) assessment of permeability classifiers. This analysis was performed between permeable (1) and impermeable (0) classifications. Feature importance was initially assessed from a model broadly trained on physicochemical metrics and the determined retention time. This model was 69 % accurate. A model trained on the metrics which gave the strongest predictive capacity from the KDE analysis; the No. hydrogen bond donors, the logD (pH = 7.40) and the retention time demonstrated improved performance of 77%. This improvement was in being able to assign impermeable drugs correctly. XGBOOST classifiers were trained from 'xgboost' python library with the following parameters and included a correction factor for population imbalances: use_label_encoder=False, eval_metric='logloss', scale_pos_weight=17/44. Analysis was performed for <800 Da drugs. Interestingly, no performance increase was observed switching No. Rotatable bonds with No. hydrogen bond donors, despite the former ranking higher in the prior, large feature number model. Finally, a model trained only on the hydrogen bond number and the retention time was 85% accurate, reflecting the results of our KDE analysis.

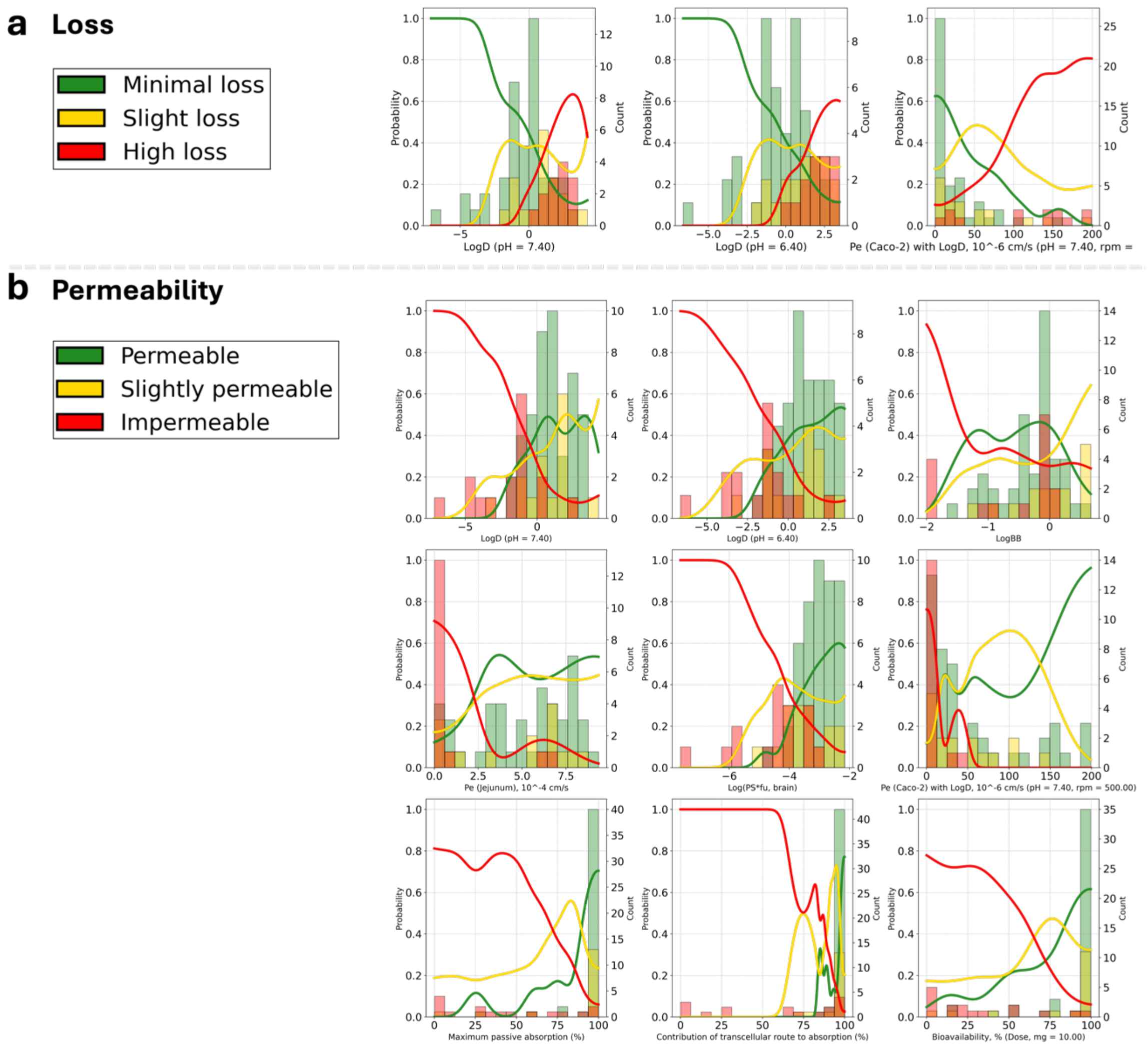

Figure S7: ADME predictors compared probabilistically to loss and permeability classifications. A series of probability distributions of ADME metrics compared across the permeability and loss classifications. Regions show probability of assignment, determined via KDE. **(a)** Across loss classifier. Note the only significant distribution separation for the loss classifier was for logD. Drugs classified minimal loss were more hydrophillic in the logD distribution. Note, that we included one drug from our hexadecane testing that exhibited over 75% signal loss to test if this would still be measurable in the DIB system (Fluoxetine HCl logD pH7.4 = 1.71). Fluoxetine could still be detected after DIB incubation and was classsified L = 1 and P = 0.5. This therefore highlights that logD was a good predictor of both permeation and loss. **(b)** Metrics detailed in Note S1. Note that for some metrics, the spread in one distribution correlated strongly to its classification, such as Caco2 and Rat Jejunem permeability rate predictions where impermeable drugs occupied a narrow spead. The inverse was true for predicted contribution to transcellular absorption and bioavailability. These correlations were upheld despite the lack of protein mediated transport mechanisms across the DIB interface.

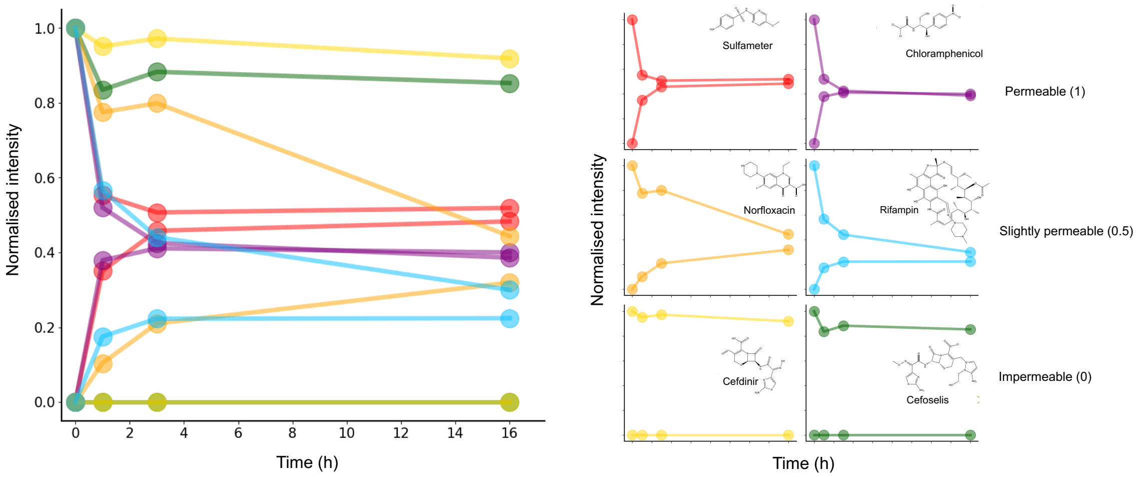

Figure S8 : Kinetic analysis of antibiotic mixture. Data determined from separating DIBs at different times and assessing peak intensity. Data shows simultaneous flux of multiple antibiotics over the bilayer with varying degrees of equilibration, confirming the equilibrium dynamics according to the classifiers.

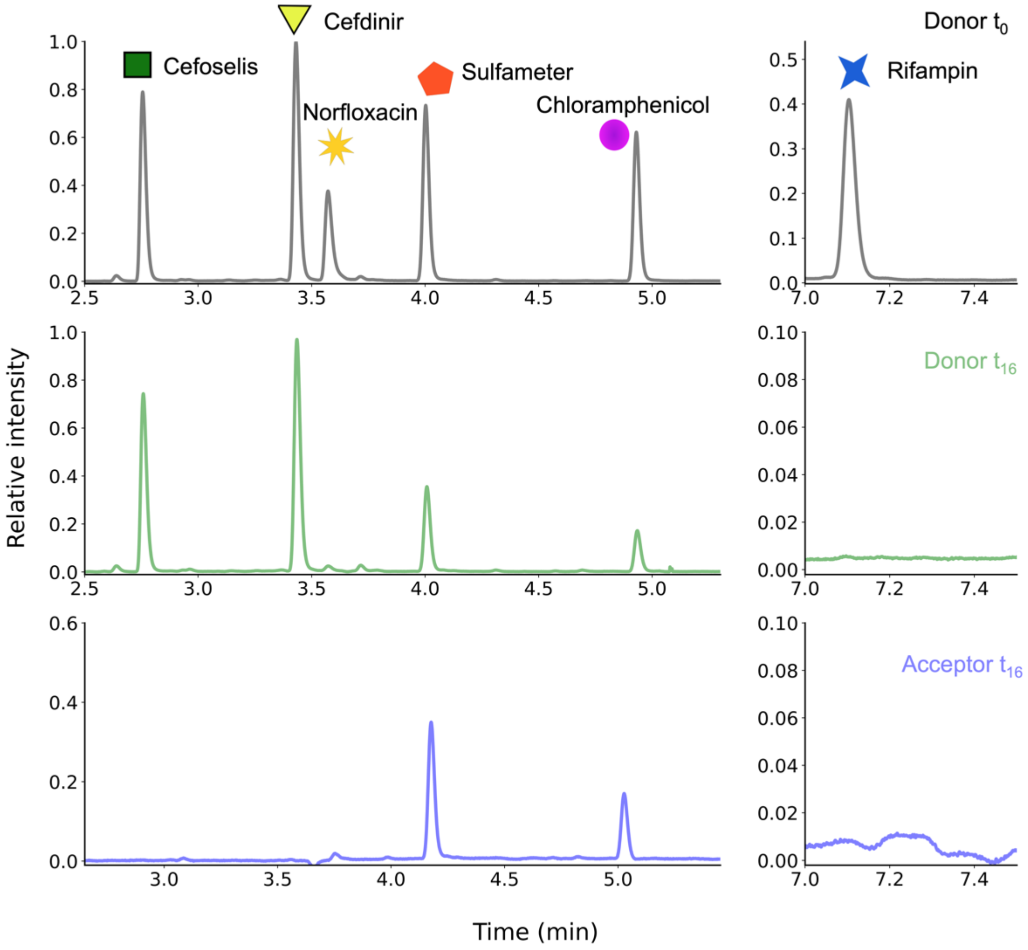

Figure S9: Antibiotic mixture analysis of passive diffusion across a reconstituted *E.coli* polar lipid extract bilayer. Data shows HPLC peak distribution. Peak heights are relative to the cefdinir peak height, t=0. Donor t0 taken from droplet composition prior to incubation at 50 °C for 1 hr followed by DIB formation and cooling to 37 °C.

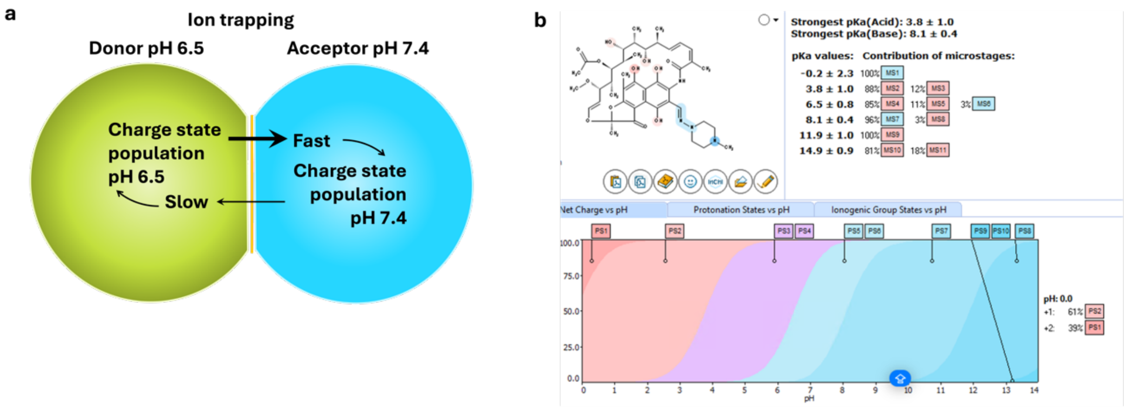

c

Antibiotic	Net charge states pH 6.5 (charge (ve) population (%))	Number of charged groups per state(count (population (%))), pH 6.5	Mean number of charged groups, pH 6.5	Total charge of ionogenic group states pH 6.5	Overall charge state pH 7.4 (charge (ve) population (%))	Number of charged groups per state (count (population (%))), pH 7.4	Mean number of charged groups, pH 7.4	Total charge of ionogenic group states, pH 7.4	Ion trapping effect strength (At=16 / D t=16 peak area)
Cefoselis	+1/-1 50%, +1 50%	2 (50 %), 3 (50 %)	2.5	0.496	+1/-1 89%, +1 11%	2 (89 %),3 (11 %)	2.11	0.107	0
Cefdinir	-2 24%, -1 76%	2 (24 %), 1 (76 %)	1.24	-1.244	-2 72%, -1 28%	2 (72 %), 1 (28%)	1.72	-1.720	0
Chloramphenicol	0 100 %	0 (100%)	0	-0.002	-1 1%, 0 99%	1 (1%), -0 (99 %)	0.01	-0.012	1.23
Sulfameter	-1 62%, 0 38 %	1 (62%) , 0 (38%)	0.62	-0.624	-1 93 %, 0 7 %	1 (93%) , 0 (7%)	0.93	-0.929	2.19
Norfloxacin	-1 1%, +1/-1 69%, +1 31%	1 (1%), 2 (69%), 1 (31%)	1.70	0.299	-1 5%, +1/-1 90 %, +1 5%	1 (5%), 2 (90%), 1 (5%)	1.90	-0.004	0
Rifampin	-2 1 %, -1 49 %, +1/-1 50 %	2 (1%), 3 (25 %) and 1(24%) 2 (50 %)	2.01	-0.450	-2 15 %, -1 75 %, +1/-1 10 %	2 (15%), 3 (38 %) and 1 (37 %), 2 (10 %)	2.01	-1.056	1.52

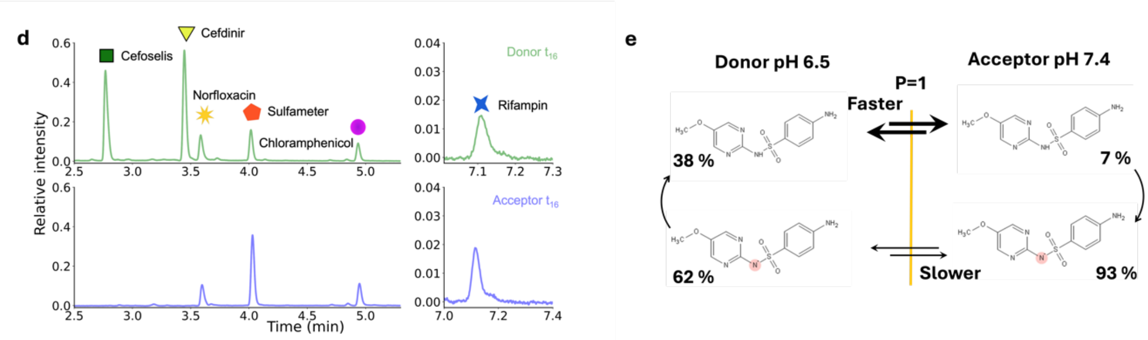

Figure S10: Ion trapping and pKa assessment of antibiotics exposed to pH gradient. (a) Cartoon highlighting pH gradient principle in DIBs for weak acids. **(b)** An example of pKa analysis for Rifampin in Percepta software. **(c)** Tabulated data of charge state populations occurring within the antibiotic mixture at the two relevant pH conditions in the DIB gradient. Charge alone was not sufficient to predict passive diffusion. Rifampin and sulfameter showed increased % of states with higher numbers of charged groups. Interestingly, across all states, rifampin had a large decrease in total charge. For sulfameter, ion trapping was significant. Critically, our assay allowed assessment of these complex dynamics occurring across a single interface. Note that the pKa distributions are considered for each in isolation, and the effect of co-solvation on individual pKa was not assessed and is outside the scope of this work. Mean ion trapping effect calculated from N=2, n = >16 experiments. **(d)** Exemplary chromatogram of ion trapping effect clearly shown for sulfameter. Relative intensity to initial donor t = 0 data. **(e)** A potential mechanism for ion trapping of sulfamater. Whilst both charge states are permeable, one state is significantly faster than the other, which drives an accumulation on the side of the membrane, as has been observed in biological systems.[5]

a

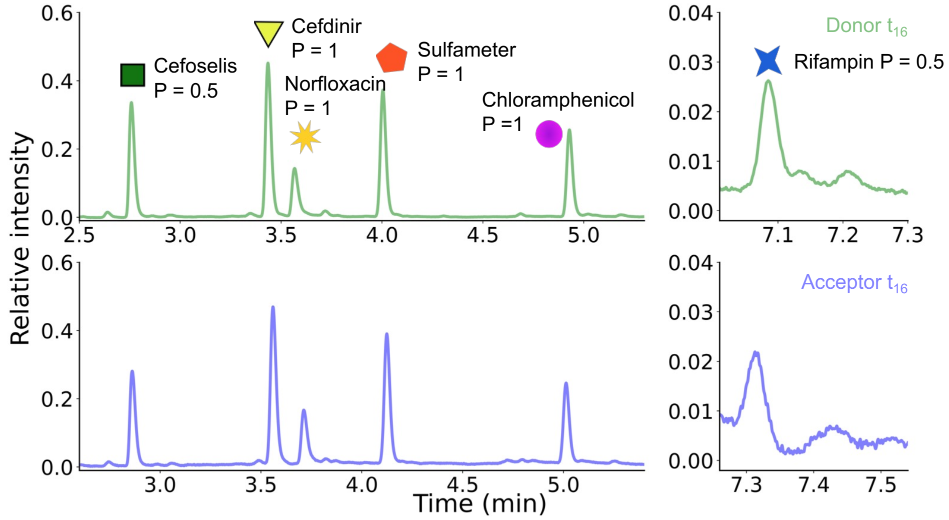

b

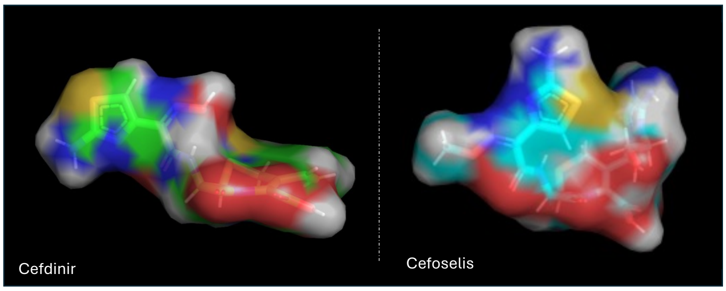

	x (nm)	x with hydration*	y (nm)	y with hydration	z (nm)	z with hydration	ΔPermClass with αHL	TPSA (Å²)
**Norfloxacin**	1.29	1.69	0.62	1.02	0.48	0.88	0.5 → 1	73
**Cefdinir**	1.52	1.92	0.66	1.06	0.47	0.87	0 → 1	211
**Cefoselis**	1.21	1.61	0.92	1.32	0.64	1.04	0 → 0.5	251
**Rifampin**	1.96	2.36	1.47	1.87	0.84	1.24	0.5 → 0.5	220

*Hydration approximated as a 4 Å increase (2 * 2 Å) in each dimension.

Figure S11: Additional investigation of passive transport experiments. (a) Data shows an additional biological repeat of the passive transport system. Peak heights are relative to the cefdinir peak height, t=0. Data labelled with permeability classification from this experiment. **(b)** Measurements of molecular bulk. Structural conformers were determined via RDKit and fed into Pymol to measure x, y, z molecular dimensions. The pore size has previously been estimated as 1.4 nm in lipid bilayers.[6]

References

1. Strutt, R., Jusková, P., Berlanda, S. F., Krämer, S. D. & Dittrich, P. S. Engineering a Biohybrid System to Link Antibiotic Efficacy to Membrane Depth in Bacterial Infections. *Small* **21**, (2025).
2. Strutt, R. *et al.* UV-DIB: label-free permeability determination using droplet interface bilayers. *Lab Chip* **22**, 972–985 (2022).
3. Bachler, S., Haidas, D., Ort, M., Duncombe, T. A. & Dittrich, P. S. Microfluidic platform enables tailored translocation and reaction cascades in nanoliter droplet networks. *Commun Biol* **3**, 769 (2020).
4. Taylor, G. J. & Sarles, S. A. Heating-enabled formation of droplet interface bilayers using escherichia coli total lipid extract. *Langmuir* **31**, 325–337 (2015).
5. Bednarczyk, D. Passive influx and ion trapping are more relevant to the cellular accumulation of highly permeable low-molecular-weight acidic drugs than is organic anion transporter 2. *Drug Metabolism and Disposition* **49**, 648–657 (2021).
6. Song, L. *et al.* Structure of Staphylococcal α-Hemolysin, a Heptameric Transmembrane Pore. *Science (1979)* **274**, 1859–1865 (1996).
